# Seasonality: The Driving Force Behind the Antimicrobial and Antioxidant Activities and Biochemical Composition of *Ericaria selaginoides* Extracts, with Minimal Effect of High Hydrostatic Pressure Pretreatment

**DOI:** 10.3390/md24080257

**Published:** 2026-07-25

**Authors:** Sunuram Ray, Maria Hortos, Andrea Casal-Silva, Mercedes Cueto, Teresa Aymerich

**Affiliations:** 1 Food Safety and Functionality Program, Institute of Agrifood Research and Technology (IRTA), 17121 Monells, Girona, Spain; sunuram.ray@irta.cat (S.R.);; 2 Institute for Integrated Studies on the Sundarbans and Coastal Ecosystems (IISSCE), Khulna University, Khulna 9208, Bangladesh; 3 Institute of Natural Products and Agrobiology (IPNA-CSIC), 38206 La Laguna, Tenerife, Spain; andreacasal@ipna.csic.es

**Keywords:** brown macroalgae, antioxidant and antimicrobial activities, high hydrostatic pressure, seasonality, pigments and polyphenol content, meroditerpenoids, *Ericaria selaginoides*, *Listeria monocytogenes*, *Bacillus cereus*, *Staphylococcus aureus*

## Abstract

The increasing interest in natural versus synthetic additives is a driving force for food industry innovation to develop alternative solutions for food safety and quality. Brown algae, described as containing potential antimicrobial and antioxidant compounds, are promising alternative sources. However, optimized food-grade extracts require preserving their bioactivity. In this study, the effects of seasonality and interannual variation, together with non-thermal high-hydrostatic-pressure (HHP) pretreatment, on the extraction of antioxidant and antimicrobial compounds from the macroalga *Ericaria selaginoides*, collected from the northwest of Spain between November 2021 and September 2023, were assessed. The HHP pretreatment of fresh algae did not significantly change the yield of the crude extracts and only slightly improved the antimicrobial activity of the extracts against *L. monocytogenes*, *S. aureus* and *B. cereus*, without significantly diminishing the antioxidant activity until 600 MPa for 5 min, the total polyphenol content (TPC), and the chlorophyll A content. Interannual and seasonal variations significantly influenced pigments and protein, carbohydrate and polyphenol contents, together with the antioxidant and antimicrobial activities, of the extracts. By NMR, phloroglucinol was identified as the major secondary metabolite, together with mannitol, alanine and a mixture of meroditerpenoids, which may contribute to the reported activities. The overall results demonstrated the role of environmental factors in driving seasonal and interannual changes in macroalgal metabolism, significantly influencing the bioactive properties of extracts, while the effect of HHP pretreatment was minimal.

## 1. Introduction

Marine bioactive compounds obtained from macroalgae have attracted increasing attention in the food sector for their role in improving food safety and quality together with health [1,2,3,4]. They have been proposed as a source of antioxidants by neutralizing free radicals and reactive oxygen species (ROSs), which are the primary initiators of lipid oxidation, thus protecting nutrients from degradation and ensuring nutritional food value over time, together with protecting health. Some studies have also suggested that macroalgae may have antimicrobial properties, which could help to reduce microbial spoilage and prevent the growth of bacteria, molds and yeasts that contribute to food spoilage and safety and health issues [5,6,7]. It was reported that as much as 25% of all food produced is lost post-harvest owing to microbial activity [8], and about 1.05 billion tons of food were wasted in 2022 [9], an alarming figure for the food security of the world. As a result, the United Nations has adopted Sustainable Development Goal (SDG) 12.3, which aims to halve global per capita food waste at the retail and consumer levels and to reduce food loss along production and supply chains by 2030 [10]. Macroalgae are increasingly being proposed as potential natural biopreservatives to be used in clean-label and plant-based food formulations to avoid or diminish synthetic chemicals and appeal to health-conscious consumers.

Food safety is still an important matter of concern worldwide. Even in the European Community, which could be considered one of the safest food markets, the number of food-poisoning-related issues is still unacceptably high [11]. *L. monocytogenes*, *B. cereus* and *S. aureus* are widely distributed in the natural environment and remain important foodborne pathogens in the EU [12,13]. *L. monocytogenes* remains one of the most concerning foodborne pathogens due to its higher mortality rate despite its relatively low incidence, as well as its ability to survive adverse conditions and contaminate ready-to-eat foods [14]. *B. cereus* continues to be a common cause of foodborne outbreaks through toxin production, reflecting its capacity to persist via heat-resistant spores in diverse food matrices, while *S. aureus* is frequently implicated in toxin-mediated outbreaks, suggesting it could survive under food-processing and -handling conditions long enough to produce enterotoxins [13]. According to the EFSA [15], these pathogens are responsible for a substantial number of food-poisoning cases, with *L. monocytogenes* causing 19 outbreaks that resulted in 133 illnesses, 84 hospitalizations, and 11 deaths. In comparison, *B. cereus* caused 474 outbreaks, accounting for 4665 illnesses, 101 hospitalizations, and four deaths, while *S. aureus* was involved in 227 outbreaks, leading to 2268 illnesses, 113 hospitalizations, and one death.

Macroalgae must survive and live within complex communities in harsh and hostile environments with a lot of competition for space, predation, and tides; thus, to cope with these ecological pressures, they have evolved to produce a wide range of complex secondary metabolites [16]. Brown algae have been described as one of the most promising sources of bioactive compounds, such as polyphenols and, particularly, phlorotannins [17,18,19]. These compounds, a family of polyphenols unique to brown macroalgae and composed of phloroglucinol units, have demonstrated significant antimicrobial and antioxidant potential. Their antimicrobial activity has been attributed to the interaction of their hydroxyl (−OH) groups with amino (−NH) groups in bacterial proteins, which may affect membrane integrity and cause cell lysis, thus inhibiting pathogen growth [20]. Consequently, phlorotannins have been proposed as natural antimicrobials and antioxidants for food, cosmetics and biomedicals.

Incorporation of marine products, especially marine algae, into an expanding variety of functional foods is suggested based on their antioxidative properties [21]. Macroalgal proteins are used in the manufacture of alternative food additives to meat patties and burgers [22]; chlorophyll A has been used as a natural pigment in food dyes [23,24]; fucoxanthin, a carotenoid with a unique chemical structure, including an allenic moiety, an epoxide group, and a hydroxyl group, has demonstrated antioxidant and antimicrobial potential [25,26,27]; and carbohydrates are used commercially as emulsifiers and thickening and gelling agents [28,29]. The meroditerpenoids isolated from *Bifurcaria galapagensis* exhibited antibacterial properties, as well as strong inhibition of cell cleavage, in fertilized eggs of the sea urchin *Stronglylocentrotus purpuratus* [30]. Nevertheless, the chemical compositions of macroalgae are significantly different among phyla, especially pigments and other secondary metabolites. They may also vary across different seasons and geographical areas of harvesting, depending on environmental factors such as temperature, solar radiation, exposure time, extraction methods, tissue age, and nutrient conditions [31,32,33]. In addition, some factors such as light, tissue age, and older tissue positions have been reported to influence fucoxanthin contents [34].

Over the past decades, various extraction techniques and solvents have been used to recover bioactive compounds, with solid–liquid extraction (SLE) being the most common [35]. Extraction efficiency depends on solvent polarity, time, and technique, with the solvent choice strongly influencing yield and bioactivity [36,37]. For food-sector applications and to ensure bioactive effects, knowledge of the presence of these bioactive compounds and their potential variability when collected in different seasons and considering other factors, together with yield extraction factors and the use of food-grade solvents, in compliance with Directive 2009/32/EC [38], is very important. In this study, n-hexane and isopropanol were used. n-hexane extracts lipophilic compounds, while isopropanol recovers moderately polar antioxidants [39,40].

Nowadays, high hydrostatic pressure (HHP) (100–1000 MPa) (a non-thermal technology used in the food industry for food safety while preserving the food’s sensorial and nutritional characteristics without changing texture and taste) has emerged as an alternative versatile technology, not only for food safety, but also to be used for optimized green extraction of natural antioxidants, while avoiding transformation/degradation of thermo-sensitive bioactive compounds from plants, food and beverages in a sustainable way [41,42]. It has been used as a substitute or complement to the use of conventional solvent-based extraction techniques like Soxhlet extraction, maceration, and hydro-distillation, thereby enabling a decrease in the drawbacks of traditional extraction, such as toxicity to humans and the environment, reducing energy and time consumption, and potential degradation of bioactive molecules during processing [43]. In brown algae, HHP has been used for assisted water extraction of alginates from *Ascophyllum nodosum* and *Saccharina latissimi* [44] and of proteins from *Fucus vesiculosus* and *Alaria esculenta* [45], with species-dependent results.

In the present study, the effects of seasonality and HHP pretreatment on the antioxidant and antimicrobial activities, as well as chemical composition, of the mid-polarity crude extracts from the fucoid *E. selaginodes*, collected from the Cantabria region of Spain during different seasons between November 2021 and September 2023, were assessed. The intended work mainly focused on the optimization of potential bioactivities to contribute as natural preservative ingredients in food. To the best of our knowledge, this is the first time research on the effects of seasonality and interannual variation, together with HHP pretreatment, to optimize extraction of bioactive compounds (antimicrobials and antioxidants) from *E. selaginoides* has been reported.

## 2. Results

### 2.1. Extraction Yield

In this study, the effect of HHP pretreatment from 55 MPa for 1 min (min) to 600 MPa for 15 min on the dry weight of *E. selaginoides* mid-polarity crude extracts was assessed in samples collected in September 2022. The pretreatment did not significantly affect (*p* > 0.05) the dry weight of the mid-polarity crude extracts obtained with a mix of isopropanol, hexane and water (80:10:10, *v*/*v*/*v*), with a mean value of 0.20 ± 0.02 g per 3.5 g of fresh algal biomass.

**Figure 1:**
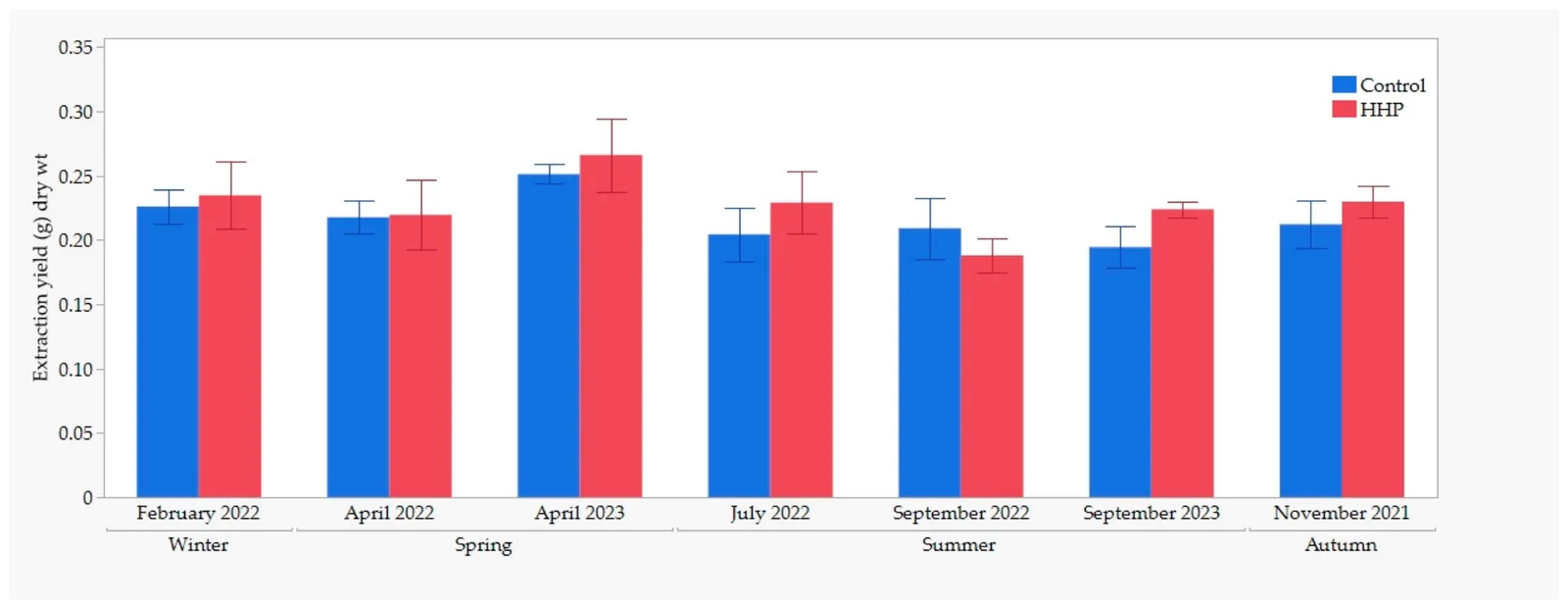
Seasonal variations in extract yield (g) of the control (fresh) and HHP-treated (600 MPa for 5 min) extracts of 3.5 g of *E. selaginoides*. Values are shown as means ± standard deviation (*n* = 3). No significance letters are shown as all groups were not statistically significant (ANOVA; Tukey’s test (*p* > 0.05)).

Subsequently, to assess the effects of seasonality and interannual variation, together with the influence of HHP pretreatment, across all collected samples from November 2021 to September 2023, HHP pretreatment at 600 MPa for 5 min was selected to compare with non-HHP-treated controls. No significant differences in extraction yield (*p* > 0.05) were obtained. The average dry weight of extracts was 0.22 ± 0.03 g (Figure 1 and Table S1).

### 2.2. Antimicrobial Activity of Extracts

The effect of HHP pretreatments (from 55 MPa for 1 min to 600 MPa for 15 min) on the antimicrobial activity of *E. selaginoides* mid-polarity crude extracts was evaluated in samples from September 2022. The results showed that fresh macroalgae pretreated with HHP (from 55 MPa for 1 min to 600 MPa for 10 min) tended to show an improved (although not statistically significant; *p* > 0.05) MIC (minimal inhibitory concentration) against *L. monocytogenes* when compared with control non-pretreated samples, as values decreased from 10 ± 5.08 mg/mL to 6.17 ± 4.07 mg/mL.

Interestingly, when lower-frequency ultrasound treatment (37 kHz) was used instead of a higher ultrasound frequency (80 kHz) for resuspension of the extracts in water–glycerol–Tween 80 (80:10:10, *v*/*v*/*v*) (WGT) buffer used for antimicrobial assays, improved activity was recorded. Indeed, the MIC values of the control samples improved from 10 ± 5.08 mg/mL to 2.18 ± 0.82 mg/mL (4.6-fold), and the average MIC values of the HHP-pretreated samples improved from 6.17 ± 4.07 mg/mL to 1.75 ± 0.61 mg/mL (3.5-fold) when a lower ultrasound frequency (37 kHz) was used for extract resuspension.

**Figure 2:**
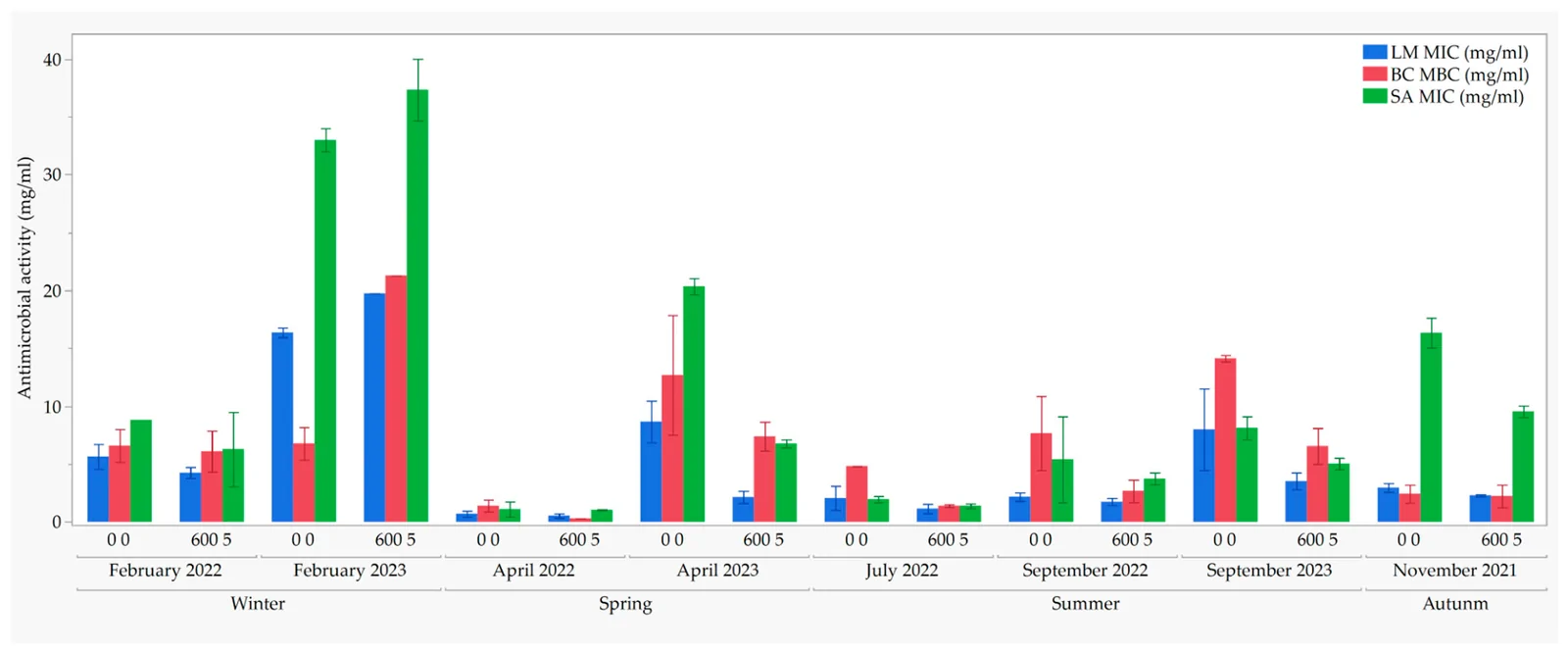
Seasonal variations in antimicrobial activity in the control (fresh) and HHP-treated (600 MPa for 5 min) extracts of *E. selaginoides*. Values are shown as means ± standard deviation (*n* = 3). No error bars indicate identical replicate values (*n* = 3). LM = *L. monocytogenes*, BC *= B. cereus*, and SA = *S. aureus*.

Thus, to further evaluate the effects of seasonality and interannual variation from November 2021 to September 2023, together with the HHP pretreatment against foodborne pathogens (*L. monocytogenes*, *B. cereus* and *S. aureus*), a pressure of 600 MPa for 5 min and a US treatment at 37 kHz were selected. The MIC values against *L. monocytogenes* and *S. aureus* and the minimum bactericidal concentration (MBC) values against *B. cereus* are represented in Figure 2. The most significant effect (see Table S2) could be attributed to month and year (*p* < 0.0001), while HHP pretreatment showed a less significant effect (*p* < 0.05) for all the foodborne pathogen targets assayed. Particularly, the HHP effect showed *p* = 0.005 for *L. monocytogenes*, *p* = 0.043 for *S. aureus* and *p* = 0.036 for *B. cereus*. HHP also produced a better correlation between the antimicrobial activities observed for *L. monocytogenes*, *B. cereus* and *S. aureus*. The observed correlation coefficients for *L. monocytogenes*–*B. cereus*, *L. monocytogenes*–*S. aureus* and *S. aureus*–*B. cereus* were r = 0.43 (*p* = 0.02), r = 0.74 (*p* = 0.0003) and r = 0.22 (*p* > 0.05) for control fresh samples and r = 0.76 (*p* < 0.0001), r = 0.78 (*p* < 0.0001) and r = 0.68 (*p* = 0.02) for HHP-pretreated samples, respectively. In general, when the effect of HHP pretreatment was evaluated across the different collection periods, the MIC values against *L. monocytogenes* and *S. aureus* decreased from 1.24- to 4.02-fold and from 1.06- to 3.00-fold, respectively, compared with the non-pretreated control extracts. The same trend was observed for *B. cereus*, with the MBC also decreasing from 1.09- to 4.62-fold when the pretreatment at 600 MPa for 5 min was applied (see Table S2). All the extracts exerted bactericidal activity against *B. cereus* and a bacteriostatic effect against *L. monocytogenes* and *S. aureus*. Thus, *B. cereus* was the most sensitive, followed by *L. monocytogenes* and *S. aureus*, as indicated by the MBC/MIC values (Figure 2 and Table S2).

The extracts exhibited the highest antimicrobial activity against the foodborne pathogen *L. monocytogenes* in 2022, especially in April 2022 (spring), with an average MIC of 0.70 ± 0.46 mg/mL. This was followed by extracts from July 2022 (early summer), with an MIC of 2.07 ± 1.46 mg/mL, and September 2022 (late summer), with 2.18 ± 0.82 mg/mL (without significant differences among them). All extracts from 2023 exhibited less antimicrobial activity when compared with 2022. Even the extracts of samples from February 2022 (winter) (MIC of 5.66 ± 3.05 mg/mL) showed slightly better activity than samples from April and September 2023, with average MIC values of 8.67 ± 3.13 mg/mL and 7.99 ± 7.04 mg/mL respectively (12.39- and 11.40-fold less antimicrobial activity than that of the extracts from April 2022). The extract with the lowest activity against this pathogen was the one from February 2023 (with an MIC of 16.38 ± 3.13 mg/mL, 23.40-fold less than that of the extract from April 2022). Concerning S. aureus, the extracts from April 2022 also exhibited the highest antimicrobial activity, with an MIC of 1.10 ± 1.14 mg/mL, followed (without significant differences) by the extract from July 2022, with an MIC of 1.97 ± 0.40 mg/mL, and that from September 2022, with an MIC of 5.42 ± 5.24 mg/mL. Also, the weakest antimicrobial effects were observed in the extracts from February and April 2023, with MIC values of 33.00 ± 1.4 mg/mL and 20.36 ± 1.20 mg/mL, respectively (30- and 18.51-fold less antimicrobial activity than in the extracts from April 2022) (Figure 2 and Table S2). For *B. cereus*, the extract obtained in April 2022 also exhibited the highest antimicrobial activity, especially in HHP-pretreated samples (MBC of 0.30 ± 0.00 mg/mL), being significantly different from samples collected in July (MBC of 1.40 ± 0.23 mg/mL) and September (MBC of 2.68 ± 1.7 mg/mL) in the same year. As for previous targets, the weakest antimicrobial effects were observed in 2023, showing MBC values of 21.28 ± 0.00 mg/mL, 7.40 ± 2.17 mg/mL, and 6.55 ± 2.67 mg/mL for HHP-pretreated samples from February, April and September 2023, respectively (70.93-, 24.67- and 21.83-fold, respectively, less antimicrobial activity than in April 2022).

Fractionation of the crude extract, performed in order to set up the active antimicrobial fraction for further compound identification, showed that only the fraction called F2 (see Materials and Methods, Section 4.3.2) reported antimicrobial activity. No antimicrobial activity was detected in the other fractions (called F1 and F3). The F2 fraction (which concentrated the antimicrobial activity of the crude extract) reported an MIC value of 0.19 ± 0.09 mg/mL against *L. monocytogenes* that was approximately 11-fold higher activity than that of the corresponding crude extract from the September 2022 sample (MIC of 2.18 ± 0.82 mg/mL).

### 2.3. Antioxidant Activity of Extracts

**Figure 3:**
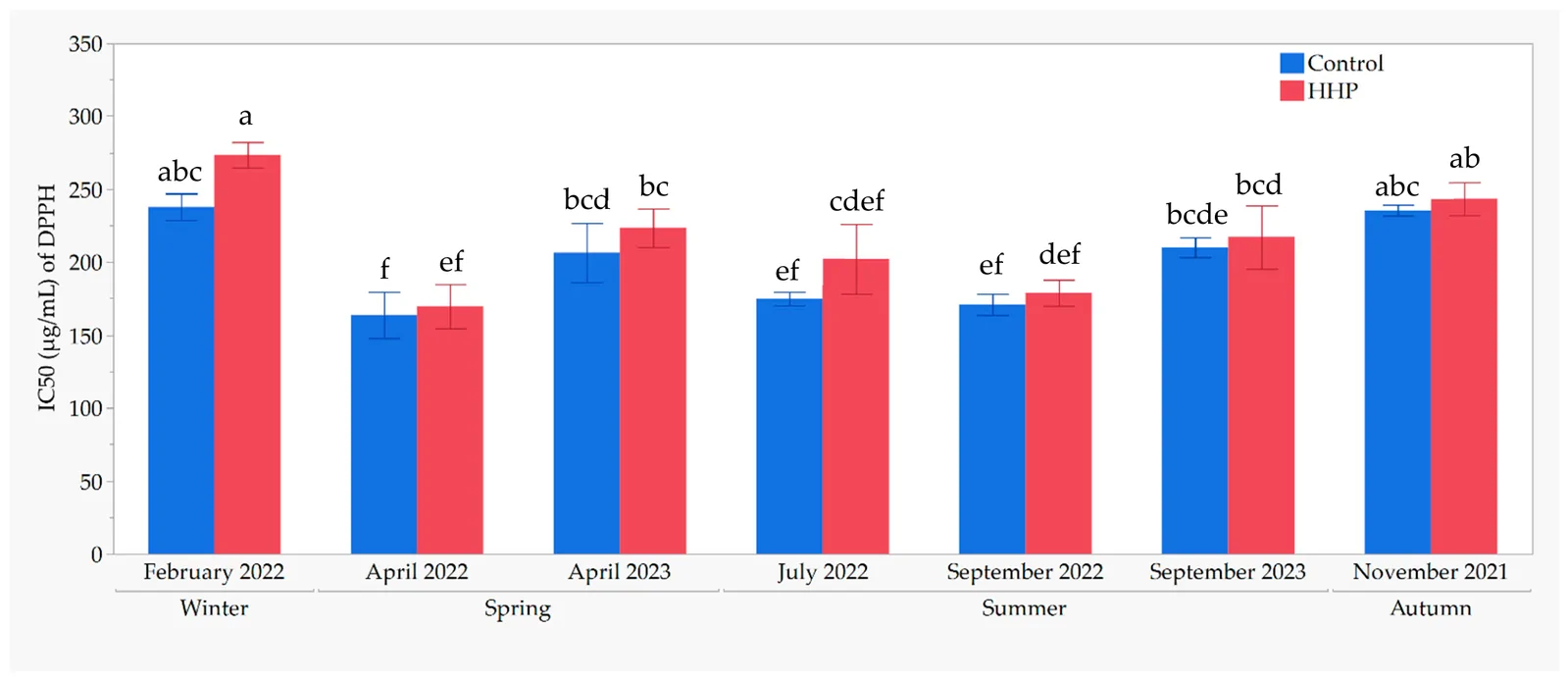
Seasonal variations in antioxidant activity (IC_50_) in the control (fresh) and HHP-treated (600 MPa for 5 min) extracts of *E. selaginoides*. Values are shown as means ± standard deviation (*n* = 3). Different letters show significance (ANOVA; Tukey’s test (*p* < 0.05)).

The antioxidant activity, expressed as the 50% inhibition concentration (IC_50_, μg/mL), exhibited significant (*p* < 0.05) seasonal and inter-annual variation over the sampling period from November 2021 to September 2023 (Figure 3 and Table S3). Concerning HHP pretreatment, the statistical analysis, comprising ANOVA with Tukey’s HSD, showed that antioxidant activity did not significantly change between the control (fresh) and HHP-treated (up to 600 MPa for 5 min) samples (*p* > 0.05). However, differently from antimicrobial activity, a significant reduction was observed at 600 MPa for 10 min (*p* < 0.05), where the antioxidant activity (IC_50_ = 272.08 ± 17.86 μg/mL) decreased by 66.23% compared with the control (IC_50_ = 163.68 ± 15.86 μg/mL) April 2022 sample.

Concerning the antioxidant activity of *E. selaginoides* extracts collected across different years and seasons, a good correlation (r = 0.81), indicating that 81% of the variation in antioxidant activity could be explained by the combined effects of month and year, was obtained. The highest antioxidant activity (*p* < 0.05) (i.e., the lowest IC_50_ values) was observed in samples collected in April 2022 (spring; IC_50_ = 163.68 ± 15.86 μg/mL) followed (without significance differences) by July 2022 (early summer; IC_50_ = 174.88 ± 4.76 μg/mL) and September 2022 (late summer; IC_50_ = 170.80 ± 7.16 μg/mL), respectively. In contrast, the highest IC_50_ was recorded in the collection period of February 2022 (winter; IC_50_ = 237.66 ± 9.25 μg/mL) and November 2021 (autumn; IC_50_ = 235.40 ± 3.72 μg/mL), respectively. When we compared the years 2022 and 2023, an inter-annual variation was found in spring samples. April 2023 recorded a significantly lower antioxidant activity (IC_50_ = 206.40 ± 20.23 μg/mL) compared with April 2022, indicating a decline in antioxidant activity in the subsequent year (Figure 3 and Table S3). Overall, the antioxidant activity showed a seasonal pattern, with significantly stronger activity between spring and summer, and weaker activity during autumn and winter. The fractionation of the crude extract from the September 2022 sample reported that the antimicrobial F2 fraction exhibited a lower antioxidant activity (IC_50_ = 811.35 ± 633.83 μg/mL) compared with the corresponding crude extract (IC_50_ = 170.80 ± 7.16 μg/mL), approximately five-fold less antioxidant activity.

### 2.4. Biochemical Compositions of Extracts

#### 2.4.1. Macronutrients (Carbohydrate and Protein)

**Figure 4:**
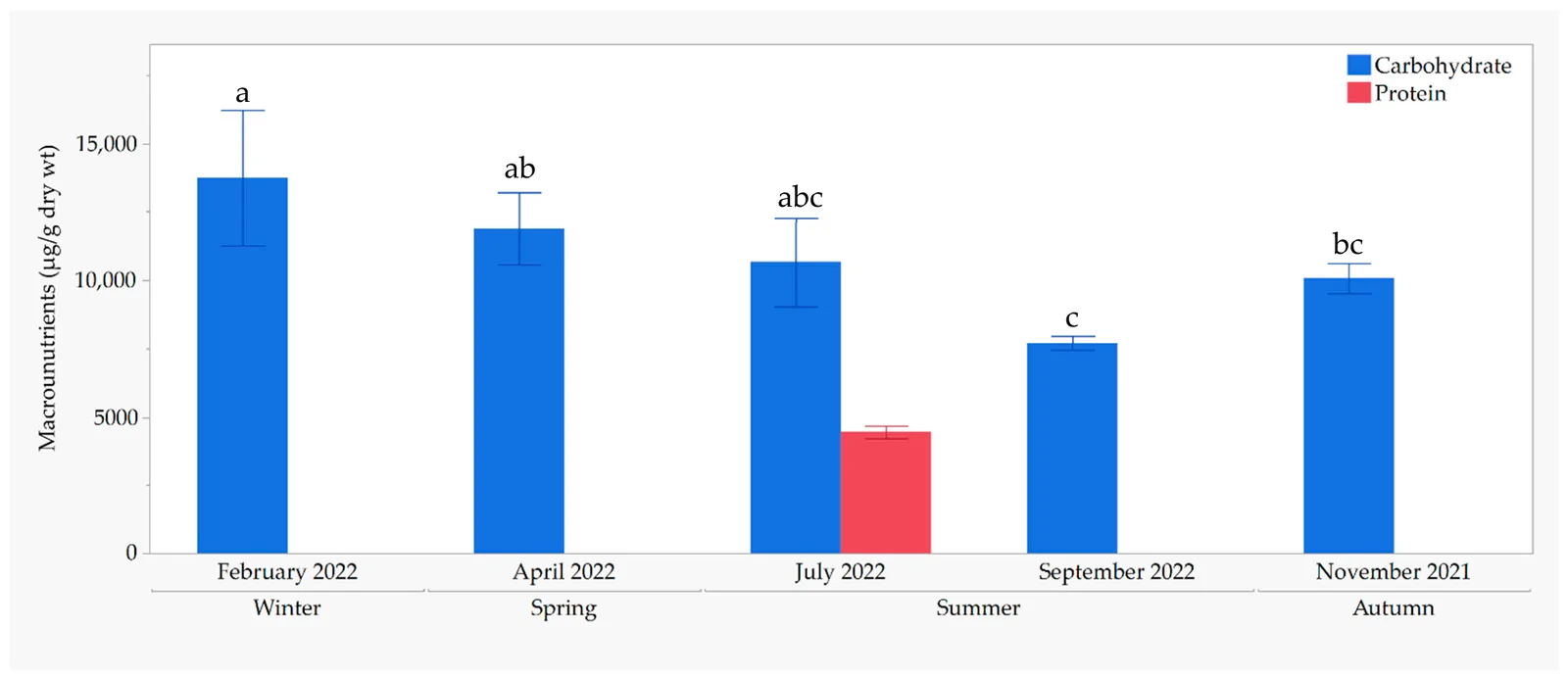
Monthly and seasonal variations in macronutrient, carbohydrate and protein contents (µg/g dry wt) in control (fresh) extracts of *E. selaginoides*. Values are shown as means ± standard deviation (*n* = 3). Significant differences (*p* < 0.05) are indicated by different superscript small letters. No red bars indicate no detection of protein.

The overall results showed that HHP did not significantly impact (*p* > 0.05) the biochemical composition (carbohydrate and protein contents) of the different pretreated samples assayed. Meanwhile, a monthly effect was observed, with a strong relation (*p* = 0.0006; R^2^ = 0.71), indicating that 71% of the variability in carbohydrate contents could be explained by the seasonal and month effect. A trend towards higher carbohydrate contents was observed in the extracts collected in February 2022 (winter), followed by April 2022 (spring) and July 2022 (early summer) (*p* > 0.05), with average values of 13,750.95 ± 2884.84 µg/g, 11,886.43 ± 1322.06 µg/g, and 10,667.55 ± 1628.19 µg/g of dried sample, respectively. The lowest content was found in September 2022 (late summer), followed by November 2021, with average values of 7716.65 ± 257.99 and 10,079.84 ± 573.47 µg/g, respectively. Indeed, the carbohydrate content tended to peak in February 2022 (winter) and was 13.56%, 22.42%, 26.70%, and 43.88% higher than the content observed in April, July, November, and September, respectively (Figure 4 and Table S4).

In the case of protein content, no effect of the HHP pretreatment was observed, while a significant seasonal and monthly effect was observed. Nevertheless, a different trend during the year was observed when compared with carbohydrate content (Figure 4 and Table S4). Protein content was only recorded in July 2022 (early summer), with an average value of 4323.69 ± 300.44 µg/g.

#### 2.4.2. Pigments (Chlorophyll A and Fucoxanthin)

**Figure 5:**
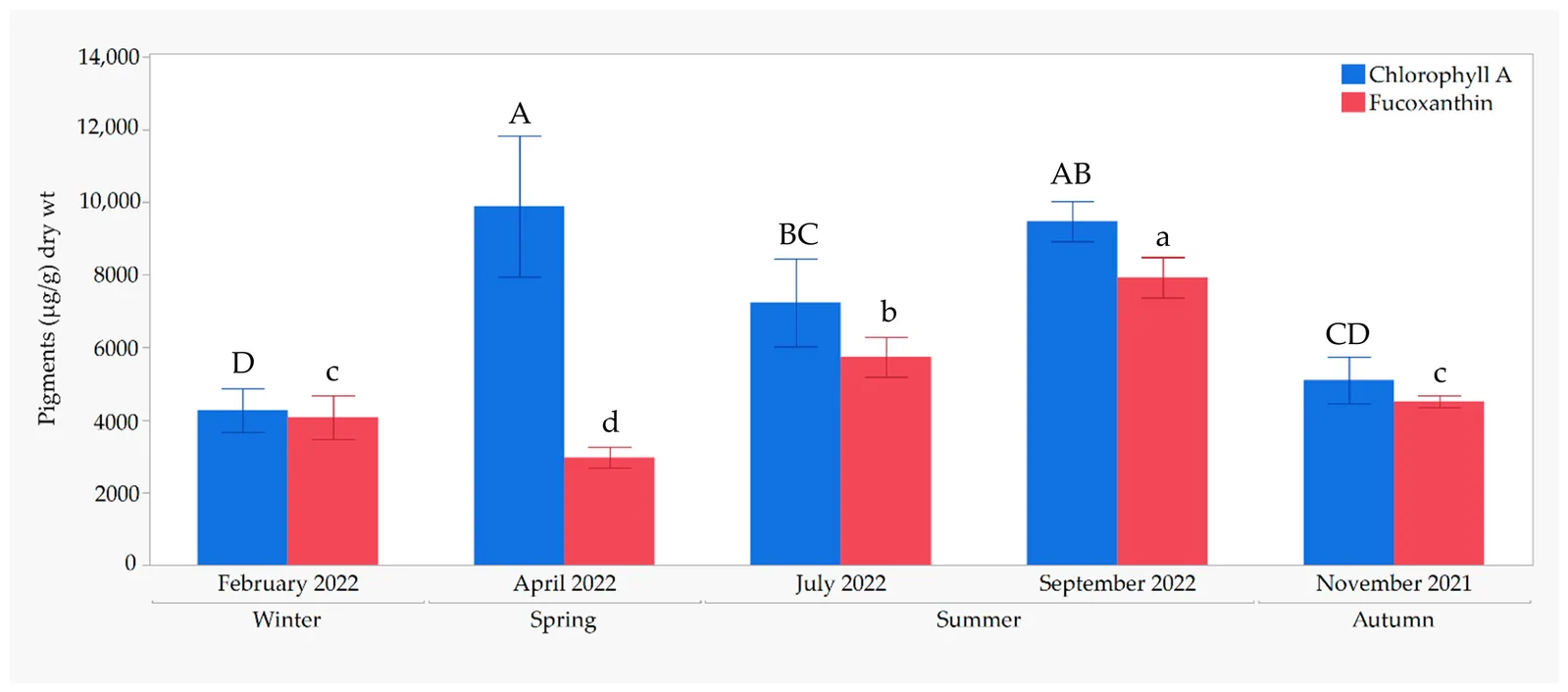
Monthly and seasonal variations in pigments, chlorophyll A and fucoxanthin contents (µg/g dry wt) in control (fresh) extracts of *E. selaginoides*. Values are shown as means ± standard deviation. (*n* = 3). Significant differences (*p* < 0.05) are indicated by different superscript capital letters for chlorophyll A and lowercase letters for fucoxanthin.

The pigments (chlorophyll A and fucoxanthin) were determined in HHP-treated and control (fresh) samples of *E. selaginoides* with different seasonality. The content of chlorophyll A of the mid-polarity extracts of *E. selaginoides* was not significantly affected by the HHP pretreatment (*p* > 0.05); nevertheless, the season and month showed a highly significant effect (*p* < 0.0001; R^2^ = 0.84). The highest content was found in April 2022 (9877.01 ± 1942.20 µg/g), followed (without significant differences) by September (9463.31 ± 549.19 µg/g) and July 2022 (7228.26 ± 1211.60 µg/g). Extracts obtained from February samples presented the lowest content of 4264.08 ± 603.18 µg/g, followed (without significant differences) by November 2021, with a value of 5095.44 ± 638.60 µg/g, indicating a notable decline (*p* < 0.05) compared with April and September (Figure 5 and Table S5).

Different from chlorophyll A and other macronutrients, the fucoxanthin content was reduced by 40% when the sample was HHP-pretreated from 300 MPa for 5 min up to 600 MPa for 10 min (without significant differences among pretreatments). The fucoxanthin content of the extracts also statistically varied with season and month (*p* < 0.0001; R^2^ = 0.95). September 2022 (late summer) showed the highest concentrations, with values of 7916.07 ± 557.20 µg/g. April 2022 (spring) presented the lowest content (*p* < 0.05), with values of 2961.46 ± 289.49 µg/g (nearly three times lower than the values obtained in September). July 2022 (earlier summer) and November 2021 (autumn) presented intermediate values, 5733.06 ± 552.80 µg/g and 4502.92 ± 171.35 µg/g, respectively (Figure 5 and Table S5).

#### 2.4.3. Bioactive Compounds

##### Total Polyphenol Content (TPC)

The effect of HHP pretreatments on the TPC of extracts was assessed. TPC followed a similar trend to that of antioxidant activity. No statistically significant differences (*p* > 0.05) were observed in TPC when control extracts from September 2022 (12,193.06 ± 678.58 µg/g dry wt) were compared with HHP-pretreated (up to 600 MPa for 5 min) samples (12,081.72 ± 432.91 µg/g dry wt). However, a significant decrease (*p* < 0.05) was observed when samples were treated at up to 600 MPa for 10 min (8938.74 ± 394.76 µg/g dry wt), resulting in a 26.66% reduction compared with the control.

**Figure 6:**
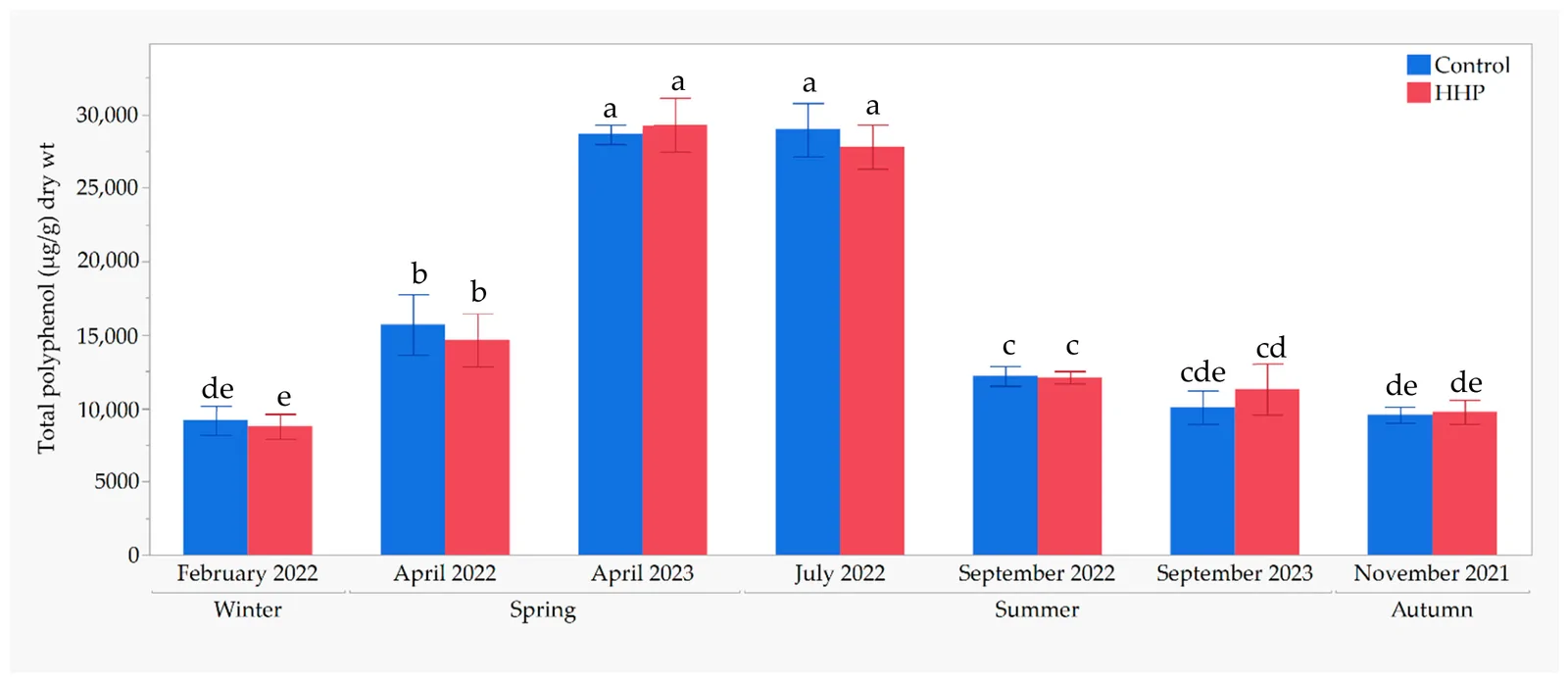
Seasonal variations in TPC in the control (fresh) and HHP-treated (600 MPa for 5 min) extracts of *E*. *selaginoides*. Values are shown as means ± standard deviation (*n* = 9). No significant difference between HPP-treated (600 MPa for 5 min) and control extracts. Different letters show significant differences (*p* < 0.05).

Given these result patterns, further analyses were carried out to investigate whether the HHP effect at 600 MPa for 5 min was maintained across all extracts obtained from samples of different years and seasons. HHP treatment showed no significant impact on TPC across different months and seasons (*p* > 0.05) (Figure 6 and Table S6). However, TPC exhibited a clear season and month trend (*p* < 0.05), with significantly higher values in early summer. In April 2022 (spring), the TPC content was 15,175.83 ± 1935.14 µg/g dry wt), while in July 2022 (early summer), it increased to 28,401.15 ± 1690.05 µg/g dry wt. An intermediate value (12,137.39 ± 555.75 µg/g dry wt) was observed in September 2022 (late summer). Lower values (8973.98 ± 945.52 µg/g dry wt and 9649.26 ± 693.74 µg/g dry wet) were recorded in February 2022 (winter) and November 2021 (autumn) respectively. It is noted that the TPC in July 2022 (summer) was about 3.16 times higher than in winter 2022 (Figure 6 and Table S6). Concerning the year, we observed an increased peak in April 2023 (spring) (28,984.35 ± 1263.76 µg/g dry wt) compared with April 2022; nevertheless, no statistical differences could be observed when September samples from both years were compared (Figure 6 and Table S6).

##### Phloroglucinol Quantification of Extract

The phloroglucinol content was quantified in a subset of samples from three different months during the year 2022: spring and mid- and late summer (April, July and September, respectively). According to the statistical Tukey test, the phloroglucinol content of the samples pretreated with HHP was not significantly different (*p* > 0.05) from that of the non-treated HHP samples, except for those that underwent the highest pretreatment at 600 MPa for 10 min. Herein, the phloroglucinol content (48.74 ± 2.38 µg/g dry wt) was significantly reduced at 600 MPa for 10 min (*p* < 0.05), when compared with the control sample (166.39 ± 30.90 µg/g dry wt). Accordingly, the same reduction trend was observed for the total polyphenol extract content at 600 MPa for 10 min (see Total Polyphenol Content (TPC).

**Figure 7:**
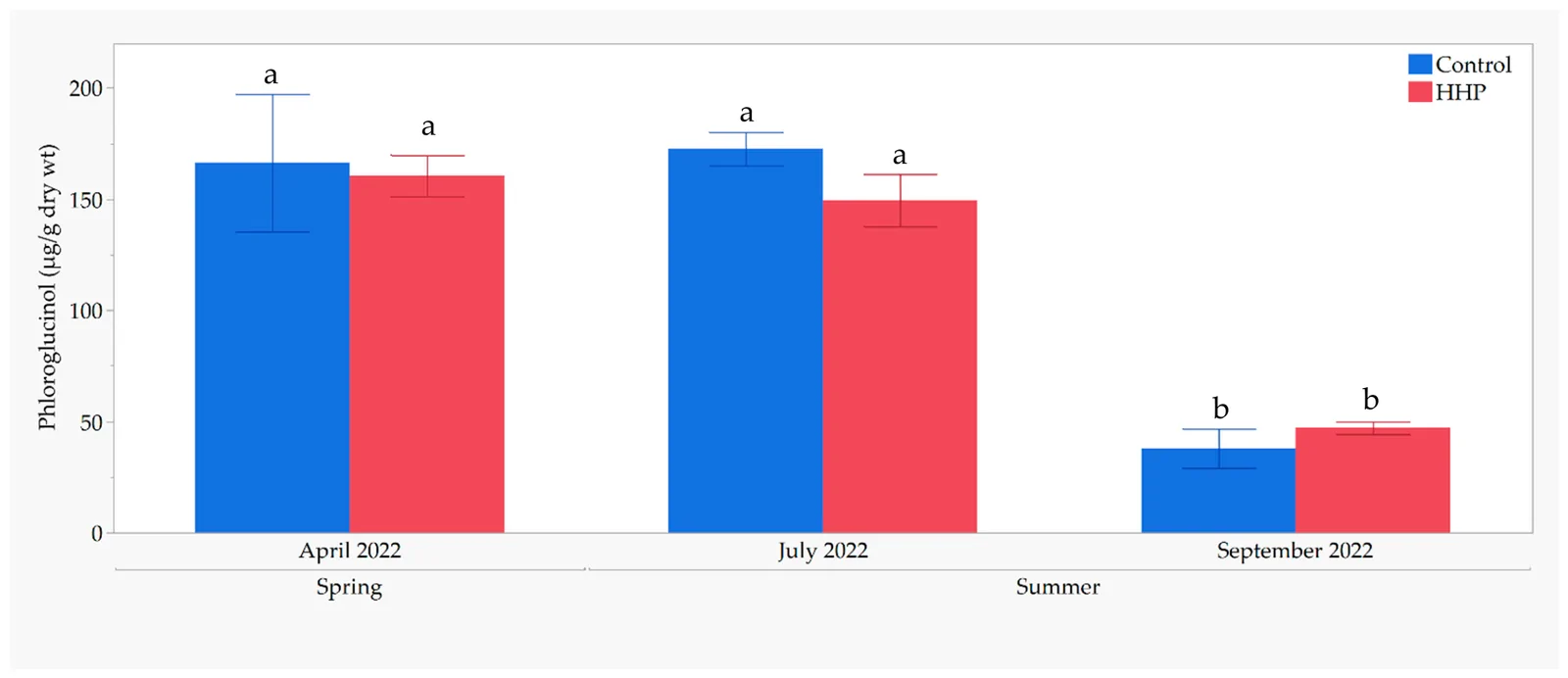
Seasonal variations in phloroglucinol contents (µg/g dry wt) in the control (fresh) and HHP-pretreated (600 MPa for 5 min) extracts of *E. selaginoides*. Values are shown as means ± standard deviation (*n* = 3). Different superscript letters show significance (*p* < 0.05).

Concerning the effect of season and month on the phloroglucinol content of *E. selaginoides*, the statistical model showed a good fit (R^2^ = 0.96) and a highly significant effect (*p* < 0.0001). This indicates that 96% of the variability in phloroglucinol content can be explained by the season and month. In our study, the extracts obtained from samples collected in April 2022 and July 2022 showed the highest phloroglucinol contents, with values of 163.53 ± 25.21 µg/g dry wt and 161.18 ± 29.75 µg/g dry wt of extract, respectively (Figure 7 and Table S7). Nevertheless, a significantly lower phloroglucinol content in the dried extract, 42.44 ± 5.85 µg/g dry wt, was observed in September 2022 (late summer) compared with April and July 2022 (*p* < 0.05). When the selected HHP pretreatment of 600 MPa for 5 min was applied to the samples collected from different periods of the year, no significant effect was observed (*p* > 0.05) when compared with the control (fresh) samples (Figure 7 and Table S7).

##### Chemical Compositions of Extracts

**Figure 8:**
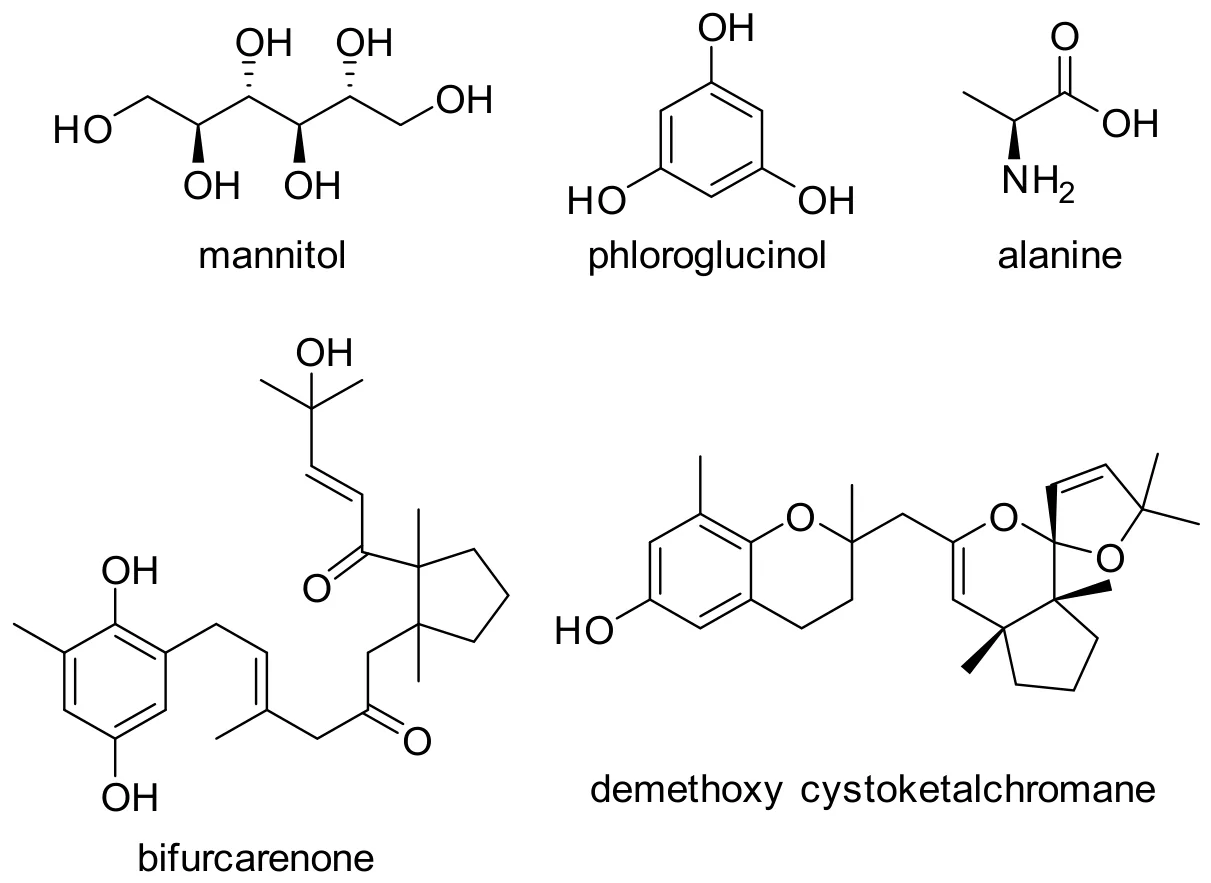
Chemical structures of the main metabolites identified by NMR.

A subset of control and HHP-treated (600 MPa for 5 min) extracts from April, July and September 2022 was further analyzed and characterized by NMR to assess the main secondary metabolite compositions. Since the *E. selaginoides* extracts did not completely dissolve in a single solvent, each extract was analyzed separately by NMR in D_2_O and CD_3_OD. Analysis of the 1D and 2D NMR spectra of all *E. selaginoides* extracts dissolved in D_2_O revealed mannitol [46] to be the major compound. Phloroglucinol [47] and the amino acid alanine [48] were also detected in all the extracts (Figure 8) (Materials and Methods, Section 4.8.6, and Supplementary Material (Figures S1–S4)).

The 1D and 2D NMR spectra of the same *E. selaginoides* extracts in CD_3_OD showed a more complex mixture of metabolites, although phloroglucinol and mannitol remained the major compounds. It is worth noting that the variations in the signal intensity of phloroglucinol between the spectra obtained in D_2_O and CD_3_OD were due to the greater solubility of phloroglucinol in methanol (Figures S5–S12).

In addition to these major metabolites, the following signals were observed: four methyl signals at δ_H_ 2.16 (3H, s), δ_H_ 1.15 (3H, s), δ_H_ 1.16 (3H, s), and δ_H_ 1.69 (3H, s) ppm; three olefinic protons at δ_H_ 6.40, δ_H_ 6.83 (d, 15.5) and 6.67 (d, 15.5) ppm; and carbon signals at δ_C_ 16.9, δ_C_ 116.2, δ_C_ 127.6, δ_C_ 146.8 ppm, δ_C_ 123.6, and δ_C_ 154.3 ppm. Also, a ketone signal was observed at δ_C_ 206.8 ppm. These signals exhibited HSQC and HMBC correlations that indicated the presence of a mixture of diterpenoids of mixed biogenesis, containing a toluhydroquinone ring similar to those of other meroditerpenoids isolated from this genus [49].

Subsequently, the F2 fractions of the *E. selaginoides* extracts from April, July and September 2022 were analyzed separately by NMR in CD_3_OD and CDCl_3_. According to the NMR analysis, they all exhibited the same characteristics.

The 1D and 2D NMR spectra of the F2 fractions from all the analyzed extracts in CD_3_OD, together with a literature comparison, allowed the identification of a major compound, the meroditerpenoid bifurcarenone. Bifurcarenone has previously been isolated from *Bifurcata galapagensis* [30] and from *Cystoseira stricta* [50], which is synonymous with *Ericaria amentacea*. Phloroglucinol was not detected by NMR in any of the F2 fractions, while mannitol was detected as a minor compound.

Analysis of the 1D and 2D NMR spectra of the F2 fractions dissolved in CDCl_3_ showed that the major metabolite present was demethoxy cystoketalchromane, which had previously been isolated from *Cystoseira amentacea* var. *stricta* [51].

Overall, the NMR data indicated that *E. selaginoides* contained a diverse profile of primary metabolites (alanine and mannitol) and phenolic and terpenoid secondary metabolites (phloroglucinol and meroditerpenoids). These findings support its potential biochemical and bioactive significance. No differences in meroditerpenoid content were observed by NMR when comparing the control and HHP-treated (600 MPa for 5 min) extracts.

Regarding the contents of the major compounds, mannitol, phloroglucinol, and alanine, in the *E. selaginoides* extracts, the distinct proton shifts of these three compounds allowed us to qualitatively analyze the relative concentration of each compound in each extract and compare the control extracts with the HHP-treated (600 MPa for 5 min) extracts. Table 1 shows the ratio of the integrals of the three phloroglucinol protons at δ_H_ 6.00 ppm with the signal at δ_H_ 3.86 ppm, which corresponds to protons H-1a and H-6a of mannitol, and the signal at δ_H_ 1.47 pp, which corresponds to the protons of the methyl group of alanine in D_2_O. For the ^1^H NMR spectra acquired in CD_3_OD, only the ratio between the integral of the signals of phloroglucinol and mannitol (δ_H_ 3.80 ppm; four protons) is shown, since the chemical shift of the alanine methyl group overlapped with other signals. The data correspond to the control and treated extracts from April and September 2022. An analysis of the values for each season (control vs. treatment) in Table 1 reveals that the phloroglucinol content decreased in September and that the HHP pretreatment (600 MPa for 5 min) did not enhance phloroglucinol extraction compared with that achieved with mannitol and alanine.

## 3. Discussion

In this study, we analyzed the antimicrobial and antioxidant activity of the crude extracts of *E. selaginoides* collected in different seasons from 2021 until 2023 and treated and non-HHP-pretreated control (fresh) samples. Beyond the evaluation of antimicrobial and antioxidant activities, we also assessed extraction yield, chemical composition such as remaining carbohydrate and protein contents, pigment contents (chlorophyll A and fucoxanthin), TPC, and secondary metabolites (phloroglucinol, mannitol, and mixed-biogenesis diterpenoids). Moreover, a further purified fraction was evaluated in order to understand the relevance of the secondary metabolites in the antimicrobial and antioxidant activity of the crude extracts. We observed that the dry weight of *E. selaginoides* mid-polarity crude extracts did not significantly vary with HHP pretreatment or with the collection period (year, season and month) (Figure 1 and Table S1). The antimicrobial activity of the extracts highly varied with the collection period and only tended to improve with the HHP pretreatment of samples (Figure 2 and Table S2). The antioxidant activity, TPC, and carbohydrate, protein, chlorophyll A, and phloroglucinol contents of the extracts varied with the collection period but not with the HHP pretreatment of samples at up to 600 MPa for 5 min (Figure 3, Figure 4, Figure 5, Figure 6 and Figure 7 and Tables S3–S7). Fucoxanthin was influenced by the collection period and HHP pretreatment at 300 MPa for 5 min. The influence of the collection period and HHP pretreatment on other secondary metabolites, such as meroditerpenoids, could not be assessed because they could not be quantified. Fractionation of selected crude extracts showed that only the F2 fraction, composed mainly of meroditerpenoids, exhibited higher antimicrobial activity and lower antioxidant activity.

### 3.1. HHP’s Effects on Yield and Antioxidant and Antimicrobial Activities

The stability of the dry matter observed in our extracts when the samples were pretreated with several HHP pretreatments partially aligned with previous studies. Tapia-Salazar et al. (2019) [52] observed no changes in the dry matter yield of 50% water/ethanol extracts from two brown algae collected between December 2014 and January 2015 from the area of Escalera, Baja California Sur (Mexico), treated at 400 MPa for 15 min. Nevertheless, when treatment at 600 MPa for 5 min was applied, a positive yield was observed in *Ecklonia arborea*, while a negative yield was seen in *Silvetia compressa*. Suwal et al. (2019) [53] reported that when HHP treatment at 400 MPa for 20 min was applied to two red macroalgae, *Palmaria palmata* (Gulf of St Lawrence, Canada) and *Solieria chorda* (from the coast of Brittany, France), no significant impact on the overall solid yield was observed when compared with non-HHP-treated samples. Rodrigues et al. (2017) [54] reported a positive impact on the yield of the water extract of the brown alga *Sargassum muticum* (collected from Buarcos Bay, Portugal, in April 2012), when a lower treatment of 300 MPa for 5 min was applied.

While the extraction yield (dry matter) of *E. selaginoides* remained largely unaffected by HHP pretreatment, slight differences emerged when evaluating the antimicrobial activity. The trend towards lower MIC/MBC values under HHP pretreatment suggests that while the extraction yield may be stable, the release, efficacy or activeness of bioactive compounds could be more prominent. It has been reported that HHP could impact cell walls by mechanical disruption and electrostatic and hydrophobic interaction changes, inducing physical, structural and conformational alterations in the structure of the algal cells, thus inducing polymer transformation and liberation from cell walls. The rupture of alginate–cellulose networks, phenolic–protein and lipid–protein complexes was reported to increase the ratio of soluble versus insoluble fiber, changing viscosity and solvent accessibility, enhancing mass transfer, and increasing solubility by hydration of charged groups in low- or non-aqueous solvents [53,55,56]. These factors could facilitate the uniform release of associated molecules, which may contribute to explaining our findings about the antimicrobial activity of the mid-polarity extracts of *E. selaginoides* pretreated with HHP. In this way, the released available antimicrobial compounds may better exert their effect against the foodborne pathogens tested.

In this study, improved antimicrobial activity was observed when a lower-frequency US treatment of 37 kHz was applied to resuspend extracts in WGT buffer. This fact was aligned with previous studies where ultrasound improved the antimicrobial efficacy of plant extracts by enhancing dispersion, bioavailability and surface contact, thus increasing interaction with bacterial target cells. Ultrasound-assisted nanoemulsion preparation of anise extract significantly increased its inhibitory effects against various pathogenic bacteria [57]. Ultrasound-assisted extraction methods for brown algae have been shown to increase the yield and bioactivity of phenolic compounds effective against *Gram-positive bacteria* [58,59]. For Gram-positive bacteria such as *L. monocytogenes*, *B. cereus*, and *S. aureus*, which possess thick peptidoglycan layers, the improved availability and diffusion of bioactive compounds likely facilitate greater disruption of cellular functions, leading to increased growth inhibition [60]. This study demonstrates that post-extraction ultrasound treatment of *E. selaginoides* extract enhanced antimicrobial activity against these Gram-positive bacterial target strains. This approach could provide an effective method to improve the solubilization and bioactivity of dried seaweed extracts, which could facilitate their potential application as biopreservatives.

In our study, antioxidant activity, TPC and phloroglucinol content remained stable under HHP pretreatment at up to 600 MPa for 5 min. To the best of our knowledge, this is the first report describing the effect of HHP on the phloroglucinol content of *E. selaginoides*. Chemically, phloroglucinol is a simple polyphenolic compound that acts as the monomeric unit of phlorotannin, thereby being closely related to TPC in brown algae [61]. The significant reduction in the antioxidant content and TPC observed after the 600 MPa pretreatment was extended to 10 min suggested that prolonged exposure may lead to chemical and enzymatic degradation of these bioactive compounds. The effect of HHP on these seaweed compounds appeared to be variable across different studies. Del Olmo et al. (2020) [62] also did not observe any effect of HHP (400 or 600 MPa for 5 min) on the antioxidant content and TPC of five different edible seaweeds (*Alaria esculenta*, *Himanthalia elongata*, *Undaria pinnatifida* (wakame), *Palmaria palmata* (dulse) and *Porphyra umbilicalis* (nori)) collected from the north of Spain. Rodrigues et al. (2017) [54] reported improved antioxidant activity of a water extract of *S. muticum* pretreated at 300 MPa for 5 min, which they attributed to the increased amount of sulfated sugar. Indeed, in our study, the carbohydrate content remained unchanged when HHP was applied. In another study, Tapia-Salazar et al. (2019) [52] reported that when two brown algae, *S. compressa* and *E. arborea*, were pretreated with HHP (400 MPa for 15 min and 600 MPa for 5 min respectively), they were affected differently, probably due to viscosity issues and interactions of the fiber with polyphenols, thus limiting their antioxidant activity. No changes in viscosity were observed in our extracts. Meanwhile, we observed that fucoxanthin was more sensitive to HHP, as its content was reduced even at a lower pressure and for a short time (300 MPa for 5 min). Mordi (1993) [63] reported that the molecule is highly sensitive to oxidation and isomerization at elevated temperatures and exposure to HHP.

### 3.2. Seasonal/Month and Yearly Variations

We found clear seasonal and interannual variations in both the antimicrobial and antioxidant activity of our extracts, with a peak in spring and summer and a marked decline in winter. This pattern is according to the results of other authors that highlighted the strong influence of environmental factors on the alga’s metabolic and defensive responses. Seasonal fluctuations in temperature, light intensity, and nutrient availability affect photosynthesis and secondary metabolism, which, in turn, determine the synthesis of bioactive compounds [64,65]. Indeed, spring and summer are characterized by intense metabolic activity and oxidative stress, triggering the enhanced production of defense-related antioxidant molecules, such as pigments, phenolics, and bioactive peptides, which may contribute to the protection of algal tissue [66,67,68] or may even contribute to inhibition of bacterial growth [69]. The colder winter months promote energy storage and reduce secondary metabolism [70]. The reproductive and vegetative stages of algae could also be important factors for plant metabolic activity and production of secondary metabolites, impacting antioxidant activity [66,67].

The antimicrobial activity followed the same seasonal trend for all three Gram-positive bacterial targets, with a peak in spring and summer. Using pooled data for both treatments at each sampling time, the best correlation was found between *L. monocytogenes* and *S. aureus* (r = 0.71; *p* < 0.001) (the extracts of the two targets’ strains exerted a bacteriostatic effect), followed by the correlation between *S. aureus* and *B. cereus* (r = 0.55; *p* = 0.0003) and *L. monocytogenes* and *B. cereus* (r = 0.41; *p* = 0.002). The extracts exerted a bactericidal effect against *B. cereus*, while a bacteriostatic effect was found against *L. monocytogenes* and *S. aureus*. These results were consistent with previous studies reporting seasonal variation in the antimicrobial potential of other brown algal extracts against different target strains, with peak activity observed during the spring season. Maréchal et al. (2004) [71] reported seasonal variation in diethyl ether (Et_2_O) extracts from the brown alga, *B. bifurcata* (collected from Roscoff, Brittany, France), with maximal inhibition (measured as the lethal concentration 50%) of *Cobetia marina* and *Pseudoalteromonas haloplanktis* during spring–summer. Similarly, the dichloromethane and dichloromethane/methanol crude extracts of *Padina pavonica* (brown alga), collected from the Northern coast of Tunisia (Cap Zebib), showed higher antimicrobial activity (zones of inhibition) in these seasons [72]. Water extracts of the alga *Grateloupia turuturu*, collected from Batz-sur-Mer (the Atlantic Coast, France), also showed the highest activity (indicated by 16% delayed growth) against *Vibrio harveyi* in spring [73].

Higher polyphenol, phloroglucinol, chlorophyll A and protein contents were found in warmer seasons, while the highest carbohydrate content was found in colder seasons. This agrees with the observations of Ford et al. (2020) [20] and Parys et al. (2009) [74] regarding total polyphenol content, phloroglucinol [75,76], chlorophyll A [39,77], protein [78] and carbohydrates [79]. The highest antioxidant activity found during warmer seasons aligns with a study on *E. selaginoides* collected from the Mediterranean Sea during 2012–2014 (La Araña Beach, Malaga, Southern Spain) [68]. The antioxidant activity followed a similar seasonal trend to antimicrobial activity, being highly correlated with the antimicrobial activity against *L. monocytogenes* (r = 0.73; *p* < 0.0001), *S. aureus* (r = 0.91; *p* < 0.0001) and *B. cereus* (r = 0.58; *p* < 0.0001).

No complete correlation between antimicrobial/antioxidant activities and any of the single compounds analyzed could be hypothesized. Total polyphenols content could only be partially correlated with antioxidant (r = 0.50; *p* = 0.0010) and antimicrobial activities against *L. monocytogenes* (r = 0.47; *p* = 0.0001) and *S. aureus* (r = 0.56; *p* = 0.0001). This partial correlation could be explained by the fact that the observed increase in the polyphenol content from April 2022 to July 2022 did not produce higher antioxidant and antimicrobial activities. These results agree with the findings of Farasat et al. (2023) [80] and Yin et al. (2015) [81] with other brown algae that found similar correlations of polyphenols with antioxidant activity (r = 0.69 and r = 0.64, respectively). Polyphenols have been reported to contribute to oxidative stress and inhibit bacterial growth [69]. Under stressful summer conditions, although TPC production is induced, their availability is simultaneously required at higher levels to support algal photoprotection; thus, molecules are transformed and oxidized, resulting in no further functional antioxidant or antimicrobial compounds remaining in the extracts. Furthermore, some good correlations were detected between phloroglucinol and both antioxidant and antimicrobial activities; however, these did not reach statistical significance (*p* > 0.05). Also, the non-complete correlation with phloroglucinol could be related to the fact that phloroglucinol is considered a phenological indicator of *C. tamariscifolia* (now known as *E. selaginoides*) to protect the receptacles at the beginning of their development in spring. Thus, the highest levels are achieved in spring, while no further increase has been observed in summer due to energy expenses needed for growth [82]. Phloroglucinol-rich extracts have been described to have a high radical-scavenging power [75,83] and strong antibacterial properties [84]. The -OH groups in phloroglucinol are likely to bind to the bacterial protein and damage the cell membranes and cell walls of bacteria, causing cytoplasm leakage and disruption of membrane permeability, and even causing cell lysis; as a result, cell death occurs [4,20,32,85]. Other authors, such as Chouh et al. (2022) [86], have reported that in *Sargassum vulgare*, phlorotannins composed of multiple phloroglucinol units have stronger antioxidant activity than simpler ones. In our study, only the small molecule was detected, and it has been observed in previous studies of this species [87].

Commercial and purified fucoxanthin from *U. pinnatifida* extract has been reported to be active against the Gram-positive and Gram-negative bacterial target strains tested [88,89], and some authors previously reported that the main reason for the antioxidant activity of seaweeds was their fucoxanthin content [90,91,92]. Nevertheless, in this study, no correlation between fucoxanthin content and antioxidant or antimicrobial activities could be found, probably due to the low content of fucoxanthin found in April 2022, when bioactivity peaks were found. These results were according to the findings of Heffernan et al. (2016) [93], who observed the highest content of fucoxanthin in summer and the lowest in spring for *F. serratus* [93]. Also, Cunningham et al. (2023) [33] reported that the fucoxanthin content may vary significantly not only between seasons but also between the months within the same season due to nutrient availability.

The higher chlorophyll A content of our extracts from April 2022 (spring) and September 2022 (summer) was in line with the results of a previous study [94,95]. A good relationship was found between chlorophyll A and antioxidant activity (r = 0.90; *p* < 0.0001) and between chlorophyll A and antimicrobial activity against *L. monocytogenes* (r = 0.83; *p* = 0.0003). Chlorophyll A has been reported to have antioxidant properties [96,97,98] and antimicrobial activity [99,100].

Protein extracts from *Saccharina longicruris* have been reported to exhibit antibacterial activity, including inhibition of *S. aureus* [101]. However, in this study, no relationship could be established between the protein contents of our mid-polarity extracts and antioxidant/antimicrobial activity, as the samples with the highest antioxidant/antimicrobial properties reported variable protein contents. Indeed, the residual protein content of the extract was only found in July (early summer). These results were consistent with the higher protein content of the brown macroalga *P. pavonica* from the Red Sea in July [78]. Concerning carbohydrate content, a negative correlation with antimicrobial and antioxidant activities was observed, as the highest content was recorded during winter (February 2022). It has been reported that algae may synthesize or store more carbohydrates under colder conditions as an energy reserve to endure harsher conditions [102], while the content is lowered in warmer, drier periods, as observed in *Fucus spiralis* from the Azores, Portugal [79].

In the crude extracts, the compounds detected by NMR, phloroglucinol and mannitol, together with alanine, and also meroditerpenoids, were commonly associated with the biological activity of brown algae. Mannitol, for example, has previously been reported to contribute to antioxidant properties [103]. Phloroglucinol, as in our study, has been reported to be the main phenolic compound of *C. tamariscifolia* [104,105], and it has been reported to exhibit both antioxidant [83] and antimicrobial properties [84]. Meroditerpenoids have also been reported to exert antioxidant and antimicrobial properties in the brown alga *Cystoseira usneoides* [106]. Bifurcarenone, one of the two meroditerpenoids found in our extracts, has been previously isolated from *Cystoseira stricta* [50], which is synonymous with *Ericaria amentacea*, and from *Bifurcaria galapagensis*, exhibiting antibacterial properties, as well as strong inhibition of cell cleavage in fertilized eggs of the sea urchin *Stronglylocentrotus purpuratus* [30].

Meroditerpenoids were also identified as the main compounds of the F2 fractionation of the crude extract where antimicrobial activity was tracked, while no phloroglucinol or chlorophyll A, and not much mannitol, was detected. This finding may confirm the relevance of meroditerpenoids as antimicrobial compounds from brown algae.

In this study, we observed that bioactive activities in our extracts were different in different years, thus suggesting that climatic interannual variability also significantly affects the production of active metabolites. This study showed that *E. selaginoides* extracts exhibited reduced bioactivity in April 2023 when compared with 2022. Taking into account the weather data report [107] in Spain, the year 2023 was the second-warmest year from 1961 until after 2022. The ocean accumulated maximum heat in 2022, and ocean temperatures surpassed, for the first time (since 1941), 20 °C during summertime. In 2023, this phenomenon of extraordinarily high temperatures was repeated. Heat waves increased. Forty-four records of warm temperatures were recorded, and, indeed, April 2023 was reported as the warmest April from the series. Furthermore, the year 2023 was reported as the driest year of the series. It has been reported that *E. selaginoides* is an alga of a temperate climate that has higher tolerance to elevated temperatures and irradiance [108]. Its growth does not begin to decline until 24 °C and does not cease completely until 28 °C [109,110]. Even some stressful conditions, such as high irradiation or high temperatures, may stimulate the production of secondary active metabolites. The high and persistent extremely increased temperatures from 2022 until 2023 may also require higher availability of algal protection. Partially transformed molecules, being that they are not further active in the extracts, could explain the lowered antioxidant/antimicrobial activity seen in 2023 when compared with the same month in 2022 [111]. As detailed by the weatherSpark report [107], spring conditions in 2022, which reported high solar irradiance, increased precipitation and wind, which also improve nutrient availability, seemed to support optimal growth and biochemical and metabolic activity to promote the accumulation of bioactive compounds in seaweeds. These findings highlight not only the seasonal but also the annual sensitivity of seaweed bioactive profiles to shifting climatic and ecological conditions, underlining the importance of monitoring environmental data across years when evaluating bioactivity.

It has to be taken into account that for further food application, the stability of the extract during food processing and storage, as well as its effects on the sensory properties of foods, should be evaluated to ensure its practical applicability as a natural food preservative [112]. Microbiological safety assessments should confirm the absence of pathogenic, antibiotic-resistant and spoilage microorganisms that could compromise food safety and quality [113]. Chemical safety issues, such as heavy metals, pesticide residues, organic pollutants, excessive iodine levels, and algal toxins, which are recognized concerns in alga-derived food products, should also be assessed [114,115], together with toxicological studies, including cytotoxicity, genotoxicity, and in vivo safety assessments, to establish safe consumption levels [112].

The overall observations of this study showed that antioxidant and antimicrobial activities obtained from crude extracts of *E. selaginoides* did not exhibit a clear single relationship with any of the bioactive compounds analyzed, such as polyphenols, carbohydrates, proteins, chlorophyll, or fucoxanthin, or the secondary metabolites detected by NMR (mannitol, phloroglucinol, alanine, and meroditerpenoids). Interestingly, fractionation of the crude extract led to a meroterpenoid-rich fraction (F2), which exhibited markedly higher antimicrobial activity but comparatively lower antioxidant activity. This suggests that antimicrobial activity in the extract may be predominantly driven by meroditerpenoids, whereas antioxidant activity is likely influenced by other compound classes that were reduced in F2, such as phloroglucinol, mannitol, and chlorophyll A. Therefore, to obtain a complete relationship, future work should aim to isolate the individual compounds and test them separately. Such further effort will allow researchers to understand the precise contribution of each compound to this issue.

## 4. Materials and Methods

### 4.1. Sample Collection

The macroalga *E. selaginoides* (Linnaeus) [116] (Ochrophyta, Phaeophyceae, Fucales, and Sargassaceae) (formerly *Carpodesmia tamariscifolia* (Hudson) Orellana and Sanson [117] and *Cystoseira tamariscifolia* (Hudson) [118]) was collected on rocky substrata in the lower intertidal and upper subtidal zones (<1 m depth) in November 2021, February 2022, April 2022, July 2022, September 2022, February 2023, April 2023 and September 2023 in Comillas (43° 23′ N/4°17′ W; the Cantabria region of Spain). The apical and median parts of the thallus of adult specimens were selected and washed in sterile seawater to remove residual sand, salt, and particles attached to their surfaces. Moreover, the remaining epiphytes and epizooties were carefully picked up. The selected material was wrapped in sterile cloths moistened with seawater and kept in darkness, maintaining a humid atmosphere (>84%) and cool conditions with ice packs (<15 °C) in expanded polystyrene (EPS) boxes Friobox S.L., Barcelona, Spain) to keep the algae alive and healthy until transport to the IRTA (Monells, Girona, Spain). In the laboratory, the algae were ground and stored in vacuum packs and protected from light at −20 °C in polyethylene terephthalate and metallic/polyethylene (PET + MET/PE) bags (oxygen permeability of 1.5 cm^2/^m^2^/24 h and water vapor permeability of 1 g/m^2^/24 h) (Sistemvac, Estudi Graf S.A., Girona, Spain) until further use.

### 4.2. High-Hydrostatic-Pressure Processing (HHP)

Fresh samples of 3.5 g packed into polyethylene terephthalate and metallic/polyethylene (PET + MET/PE) bags (oxygen permeability of 1.5 cm^2/^m^2^/24 h and water vapor permeability of 1 g/m^2^/24 h) (Sistemvac, Estudi Graf S.A., Girona, Spain) were used for HHP pretreatment. The HHP pretreatments of the macroalgae were performed in an industrial pressurization unit (Wave 6000, NC Hyperbaric, Burgos, Spain). Fresh macroalgal samples of *E. selaginoides* collected from April and/or September 2022 (late summer) were treated under various pressure and time conditions, including 55 MPa for 1 min, 100 MPa for 2.5 min, 200 MPa for 2.5 min, 300 MPa for 2.5 min, 300 MPa for 5 min, 300 MPa for 10 min, 600 MPa for 5 min, 600 MPa for 10 min and 600 MPa for 15 min, in order to assess the effect of pressure and time on the pretreated samples. As no statistically significant differences were seen among the pretreatments at up to 600 MPa for 5 min, the highest pretreatment preserving bioactivities was selected to compare the effect of HHP on samples from different seasons and years, such as those from November 2021, February 2022, July 2022, February 2023, April 2023 and September 2023. The selection of 600 MPa for 5 min was driven by food safety considerations, ensuring effective microbial control prior to further processing while keeping bioactivity. The pressurization fluid was water, the come-up time was 6.8 min, the pressure release was for 0.8 min, and the temperature was set to 10 °C.

### 4.3. Preparation of Macroalgal Extracts

#### 4.3.1. Chemical Extraction Reagents

Hexane–acetone (HPLC-grade) was obtained from VWR (VWR International Eurolab S.L., Barcelona, Spain) and isopropanol (2-propanol) from Merck (Merck Life Science S.L.U., Madrid, Spain). Ultrapure water was obtained by a Milli-Q system (Millipore, Merck Life Science S.L.U., Madrid, Spain). In line with current regulations and safety considerations regarding the use of natural solvents for producing macroalgal extracts with potential food applications, food-grade solvents were selected for this study. All the solvent chemicals were of analytical grade and according to the rules of Directive 2009/32/EC [38], as well as the current ICH guideline for industry ICH Q3C of the Food and Drug Administration (FDA) (2-propanol, class 3, and hexane, class 2). Also, residual levels were minimized to meet legal limits, ensuring safety.

#### 4.3.2. Extraction Procedure

Crude mid-polarity extracts from 3.5 g of fresh algal samples, pretreated or not pretreated with HHP, were prepared using 90 mL of an extraction medium composed of a mixture of E10 (hexane–isopropanol–water (10:80:10, *v*/*v*/*v*). Amber wide-mouth high-density polyethylene (HDPE) bottles (VWR International Eurolab S.L.U., Barcelona, Spain) of 250 mL were used [119]. The pooled supernatants (5000 rpm, 10 min, and 4 °C; Eppendorf, Hamburg, Germany) of three consecutive extractions performed at room temperature for 30 min each were evaporated to dryness under vacuum conditions at room temperature (Rotovapor^®^-R 114, BUCHI Ibérica S.L.U., Barcelona, Spain, and Thermo Scientific™ Savant™ Speed Vac™ SPD120 Vacuum Concentrator, Fisher Scientific S.L., Madrid, Spain). The dried extracts were collected, weighed, and kept frozen at −20 °C until further analysis. Moreover, in order to identify the potential compounds responsible for the antimicrobial activity of the crude extracts, we further purified the crude extracts by solvent fractionation. First, the crude extract in E10 was mixed 1:1, *v*/*v* with miliQ water. Then, the upper fraction (F1) containing chlorophyll A was removed, and the bottom phase was mixed 2:1, *v*/*v* with n-hexane. After 18 h in darkness, the upper phase (F2) and bottom phase (F3) were collected separately, dried and kept until further use.

### 4.4. Antimicrobial Activity Screening

For antimicrobial activity, extract residues were dissolved under sterile conditions in 1 mL of an innocuous buffer for bacterial growth, composed of a mixture of water–glycerol–Tween 80 (80:10:10, *v*/*v*/*v*) (WGT). Two ultrasound treatments of extracts were used (80 and 37 kHz for 15–30 min) (controlling the temperature with ice water) in order to assess their contribution to the solubility and homogeneity of the potential antimicrobial active compounds of the extracts.

### 4.5. Test Microorganism and Culture Conditions

The antimicrobial activity was tested against the Gram-positive bacteria *L. monocytogenes* CTC1011 (IRTA-Monells), *B. cereus* LMG12335 and *S. aureus* CECT976. The strain was cultivated from a −80 °C stock culture cryoprotected with 20% glycerol (*v*/*v*) during 18 h in TSBYE (Tryptic soy broth, VWR International Eurolab S.L., Barcelona, Spain) supplemented with 0.6% Bacto^®^ yeast extract (BD Biosciences, Fisher Scientific S.L., Madrid, Spain) during 24 h at 37 °C prior to assay.

### 4.6. Determination of MIC (Minimum Inhibitory Concentration) and MBC (Minimum Bactericidal Concentration)

The MIC and MBC were assayed for every active extract using the microdilution method in a 100-well plate according to the criteria recommended by the Clinical and Laboratory Standards Institute with modifications [120]. An amount of 200 µL of 24 h bacterial cultures grown in BHI (GranuCult™ Brain Heart Infusion Broth, Merck, Merck Life Science S.L.U., Madrid, Spain) at the optimal growth temperature for each strain was added to the microtiter plate wells to have a final bacterial concentration of 10^9^ cfu/mL in each well. Row A was referred to as the growth control for each of the assayed strains. In row B, 400 µL plus 20 µL of each extract were added, and serial two-fold dilutions were carried out in order to measure the antibacterial activity. Assays were performed in duplicate for each strain. Tests were performed in 100-well plates at 37 °C for 18 to 24 h. Growth was quantified by optical density (OD) measurement at 420–580 nm using Bioscreen C (Bioscreen, Thermo Fisher Scientific S.L.U, Madrid, Spain). The minimum concentration that inhibited the growth of the bacteria was determined as the MIC. The results were confirmed by TSAYE plate counts. The MBC was set up at a 3-log reduction from the original inoculum in the microtiter wells.

### 4.7. Antioxidant Activity Evaluation Through DPPH Free Radical Scavenging Assay

The antioxidant activity of the sample was determined using a 2.2-diphenyl-1-picrylhydrazyl (DPPH, Merck Life Science S.L.U., Madrid, Spain) free radical assay and following the method described by [121]. A calibration curve with a set of 10 known concentrations of Trolox (0, 0.01, 0.02, 0.02, 0.04, 0.05, 0.06, 0,07, 0.08 to 0.09 mM) was prepared. All the prepared reactions were incubated for 40 min in darkness at room temperature. The absorbance of each of the incubated solutions was determined at 517 nm using a Varioskan, GENESYS™ 10S UV-Vis spectrophotometer (Thermo Fisher Scientific S.L.U. Madrid, Spain) against a blank. A set of 10 dilutions (1:2, *v*/*v*) for each sample was prepared and assayed. Analysis was done in triplicate. The radical-scavenging activity was calculated, taking into account the following formula:

% Inhibition rate: {(A _control_ − A _sample_)/(A _control_)} × 100.

where A _control_ represents the absorbance of the DDPH solution without the sample and minus the A _control blank_, and A _sample_ represents the absorbance of the sample minus the A _sample blank_. The results were expressed in µg/mL and mmol of Trolox equivalents (Trolox Eq)/mg dry weight of seaweed. The IC_50_ was calculated from the optimized ranges of the sample encompassing the 50% scavenging concentration for the DPPH radical, considering the regression line within the linear segment of the curve and using logistic regression 5P (JMP^®^ Student Edition 19, 2025, JMP Statistical Discovery, LLC., Cary, NC, USA) with a sigmoidal model for the complete curve. The correlation between both models was R^2^ = 0.994 (*n* = 87).

### 4.8. Determination of Chemical Composition

The characterization of the TPC and chlorophyll A, fucoxanthin, carbohydrates, protein, phloroglucinol and other compound contents was assessed in HHP-treated and non-treated samples from different seasons and year collections.

#### 4.8.1. Carbohydrate Content

The total carbohydrate content was evaluated by the phenol–sulfuric acid method [122,123]. A dried 200 µL aliquot of the extract, performed in triplicate, was resuspended with 200 µL of water, and then 200 µL of 5% phenol (Sigma-Aldrich, Merck Life Science S.L.U., Madrid, Spain) and 1 mL of sulfuric acid at 96% (Panreac, Barcelona, Spain) were added and mixed with the residue. The sample was incubated for 15 min at 30 °C, and a 250 µL aliquot was transferred to a microtiter plate. The absorbance was read at 490 nm (Varioskan spectrophotometer). A calibration curve with 25–250 ng/µL of glucose was generated in triplicate. The results were expressed in micrograms of glucose equivalents per gram of dry weight of seaweed (µg/g dry wt).

#### 4.8.2. Protein Content

The total protein content of the samples was estimated spectrophotometrically according to the Bradford method [124]. To avoid interference with the extraction media during the reaction, 200 µL of the extract was first dried with nitrogen in a 2 mL conical tube and then resuspended in the same amount of water. Each sample was mixed with 1 mL of Bradford reagent diluted (1:6, *v*/*v*) with water (Bio-Rad Protein Assay; Bio Rad Laboratories S.A., Madrid, Spain), and the mixture was left to stand for 30 min at room temperature. The absorbance was measured at 595 nm using the Varioskan spectrophotometer after 250 µL was placed into a microtiter plate. Each standard and sample solution was run in triplicate. Bovine serum albumin (BSA) (Sigma-Aldrich, Merck Life Science S.L.U., Madrid, Spain) was utilized as the standard, and values ranging from 25 to 200 ng/µL were employed to create a calibration curve. The results were reported as micrograms of BSA equivalents per gram of seaweed dry weight (µg/g dry wt).

#### 4.8.3. Pigment Content

The pigment content was determined by HPLC measurement, as reported by Rubiño et al. (2022) [123]. For HPLC measurement, all extracts were previously filtered (0.22 µm PTFE), and an aliquot (10–25 µL) was injected into the HPLC system (Agilent Technologies Spain S.L., Barcelona, Spain). Pigment separation was performed in a Kinetec EVO C18 column (5 µm; 250 × 4.6 mm) (Phenomenex, España S.L.U., Madrid, Spain) at 25 °C in a gradient mode, from water (phase A) to 98% acetonitrile–isopropanol (50:50, *v*/*v*, phase B). The gradient elution was as follows: 5 min from 25% to 55% B; 20 min from 55% to 98% B; 5 min from 98% to 25% B; and 3 min at 25% B. The elution rate was set at 1 mL/min. The detection was performed at 666 nm for chlorophyll A and 434 nm for fucoxanthin, respectively. For reference materials, stock solutions of commercial pigments, fucoxanthin and chlorophyll, from Sigma-Aldrich (Merck Life Science S.L.U., Madrid, Spain), were used for linearity at 20 ng/µL.

#### 4.8.4. Total Phenol Content (TPC)

The TPC was determined using the Folin–Ciocalteu reagent (FCR) adapted to the microtiter plate measurement [119,125]. In summary, an aliquot (100 µL) of the extract was mixed with 500 µL of the Folin–Ciocalteu reagent (Sigma-Aldrick, Merck Life Science S.L.U., Madrid, Spain) diluted to 1:10 (*v*/*v*) with ultrapure water and 400 µL of a 12.5% sodium carbonate solution (Sigma-Aldrich, Merck Life Science S.L.U., Madrid, Spain). The mixture was incubated for 15 min at 45 °C and centrifuged at 5500 rpm for 5 min. An amount of 250 µL of the samples, performed in triplicate, was transferred to a microtiter plate, and the absorbance was read at 765 nm with the Varioskan spectrophotometer. A calibration curve in the range of 10–80 ng/µL was generated with phloroglucinol (Sigma-Aldrich, Merck Life Science S.L.U., Madrid, Spain) in triplicate. The results were expressed as micrograms of phloroglucinol equivalents (PGE) per gram of seaweed dry weight (µg/g dry wt).

#### 4.8.5. Quantification of Phloroglucinol Content by HPLC-MS

The phloroglucinol content of *E. selaginoides* extract was determined using an integrated HPLC-MS. The samples were prepared by dissolving 1 mg of dried extract in 1 mL of methanol, filtering them with a 0.2 μm porous filter, and used for analysis. HPLC/ESI-MS analysis was conducted using a Waters VION IMS QTof (Waters Corporation, Altrincham, UK). Electrospray (ESI) was employed as the ion source, operating in positive (ESI (+)) ion mode and the operation conditions were source temperature: 120 °C; desolvation temperature: 600 °C; cone gas: 50 L/h; desolvation gas: 1000 L/h; capillary: 0.80 kV; sample cone voltage: 40 V; and source offset voltage: 80 V. Mass spectra were recorded in the range of *m*/*z* 50–1000. An ACQUITY UPLC^®^ BEH C18 1.7 μm (Waters Cromatografia S.A., Barcelona, Spain) was used as the stationary phase, while the mobile phase was composed of water (A) and 0.1% formic acid and MeOH and 0.1 HCOOH (B) (Sigma-Aldrich, Merck Life Science S.L.U., Madrid, Spain). The gradient was as follows: 0–0.5 min, 100% A; 1 min, 90% A; 3 min, 90% A; 3.8 min, 50% A; and 5 min, 5% A. The re-equilibration time was 4 min. The flow rate was 300 μL/min. The injection volume was 2 μL. For quantitative analysis, five concentrations of phloroglucinol (0.1, 1, 10, 20 and 50 μg/L) were prepared in HPLC-grade methanol and were injected in triplicate. Extract samples of 1 mg/mL were prepared. The standard curves were prepared by plotting the average of the peak areas versus the concentrations of each compound. The linearity of each calibration curve was assessed by linear regression analysis, which was calculated by the least-squares method.

#### 4.8.6. Secondary Metabolites Determined by NMR Analysis of Extracts

^1^H NMR, ^13^C NMR, HSQC, HMBC and COSY spectra were measured, employing a Bruker AMX 500 instrument (Bruker Karlsruhe, Germany) operating at 500 MHz for ^1^H NMR and at 125 MHz for ^13^C NMR. All ^13^C and ^1^H NMR spectra were internally referenced to the residual solvent signal (CDCl_3_: δ_C_ 77.0 ppm, δ_H_ 7.25 ppm; CD_3_OD: δ_C_ 49.0 ppm, δ_H_ 3.31 ppm; or D_2_O: δ_C_ 0.0 ppm (internal reference 3-(trimethylsilyl)propionic-2,2,3,3-d_4_ acid sodium salt), δ_H_ 4.79 ppm). Two-dimensional NMR spectra were obtained using TopSpin 4.4.0 software.

Phloroglucinol: ^1^H (500 MHz, D_2_O) δ 6.00 (3H, s); ^13^C NMR (125 MHz D_2_O) δ 95.2 (CH), 157.6 (C). ^1^H (500 MHz, CD_3_OD) δ 5.79 (3H, s); ^13^C NMR (125 MHz CD_3_OD) δ 95.5 (CH), (160.1, C).

Mannitol: ^1^H (500 MHz, D_2_O) δ 3.86 (2H, *dd*, 2.6, 11.8, H-1a, H-6a), 3.79 (2H, *m*, H-2, H-5), 3.75 (2H, *m*, H-3, H-4), 3.67 (2H, *dd*, 6.8, 11.8, H-1b, H-6b); ^13^C NMR (125 MHz D_2_O) δ 63.2 (CH_2_, C-1, C-6), 69.2 (CH, C-2, C-5), 70.8 (CH, C-3, C-4); ^1^H (500 MHz, CD_3_OD) δ 3.81 (2H, *dd*, 3.4, 11.2, H-1a, H-6a), 3.80 (2H, *m*, H-2, H-5), 3.70 (2H, *m*; H-3, H-4), 3.65 (2H, *dd*, 5.9, 11.2, H-1b, H-6b) ^13^C NMR (125 MHz CD_3_OD) δ 65.0 (CH_2_, C-1, C-6), 71.2 (CH, C-2, C-5), 72.9 (CH, C-3, C-4).

Alanine: ^1^H (500 MHz, D_2_O) δ 1.52 (3H, *d*, 7.3), 3.79 (1H, *m*); ^13^C NMR (125 MHz D_2_O) δ 16.1 (CH_3_), 50.5 (CH), 175.8 (C); ^1^H (500 MHz, CD_3_OD) δ 1.49 (3H, *d*, 7.3), 3.66 (1H, *m*); ^13^C NMR (125 MHz CD_3_OD) δ 17.2 (CH_3_), 51.9 (CH), 176.6 (C).

Bifurcarenone: ^1^H (500 MHz, CD_3_OD) δ 1.16 (3H, s, H-19), 1.17 (3H, *s*, H-18), 1.29 (3H, *s*, H-17), 1.30 (3H, *s*, H-16), 1.53 (1H, m, H-10), 1.67 (3H, *s*, H-20), 1.71 (3H, *m*, H-8, H_2_-9,), 1.90 (1H, *m*, H-8), 2.16 (3H, *s*, 6′-Me), 2.27 (1H, *m*, H-10), 2.30 (1H, *d*, 15.7, H-6), 2.46 (1H, *d*, 15.7, H-6), 3.06 (2H, *s*, H-4), 3.29 (2H, *s*, H-1), 5.41 (1H, *ddd*, 1.0, 7.3, 7.3, H-2), 6.38 (1H, *d*, 2.7, H-5′), 6.40 (1H, *d*, 2.7, H-3′), 6.67 (1H, *d*, 14.9, H-13), 6.83 (1H, *d*, 14.9, H-14); ^13^C NMR (125 MHz CD_3_OD) δ 16.6 (CH_3_, C-20), 16.9 (CH_3_, 6′-Me), 20.6 (CH_3_, C-18), 20.8 (CH_3_, C-16), 20.9 (CH_2_, C-9), 21.5 (CH_3_, C-19), 27.5 (CH_3_, C-17), 29.5 (CH_3_, C-16), 29.9 (CH_2_, C-1), 35.3 (CH_2_, C-10), 37.7 (CH_2_, C-8), 47.9 (C, C-7), 48.1 (CH_2_, C-6), 56.8 (CH_2_, C-4), 61.3 (C, C-11), 71.5 (C, C-15), 114.5 (CH, C-5′), 115.9 (CH, C-3′), 124.0 (CH, C-13), 127.8 (C, C-6′), 129.5 (CH, C-2), 130.6 (C, C-3), 130.9 (C, C-2′), 146.5 (C, C-1′), 151.4 (C, C-4′), 154.7 (CH, C-14), 206.7 (C, C-12), 211.4 (C, C-5).

Demethoxy cystoketalchromane: ^1^H (500 MHz, CDCl_3_) δ 0.88 (3H, *s*, H-19), 1.13 (3H, *s*, H-18), 1.28 (3H, *s*, H-17), 1.32 (3H, *s*, H-20), 1.35 (3H, *s*, H-16), 1.55 (2H, *m*, H-9), 1.67 (2H, *m*, H-8), 1.76 (2H, *m*, H-2), 2.25 (2H, *m*, H-4), 2.71 (2H, *m*, H-1), 4.35 (1H, *brs*, H-6), 6.06 (1H, *d*, 5.6, H-13), 5.58 (1H, *d*, 5.6, H-14), 6.37 (1H, *brs*, H3′), 6.46 (1H, *brs*, H5′); ^13^C NMR (125 MHz CDCl_3_) δ 16.2 (CH_3_, Me-6′), 20.3 (CH_3_, C-19), 20.9 (CH_2_, C-9), 22.7 (CH_2_, C-1), 22.6 (CH_3_, C-18), 25.3 (CH_3_, C-20), 26.7 (CH_3_, C-17), 29.0 (CH_3_, C-16), 31.1 (CH_2_, C-2), 35.9 (CH_2_, C-10), 40.9 (CH_2_, C-8), 43.2 (C, C-7), 44.2 (CH_2_, C-4), 46.2 (C, C-11), 75.8 (C, C-3), 88.5 (C, C-15), 111.2 (CH, C-6), 115.2 (C, C-12), 115.7 (CH, C-5′), 121.5 (C, C-2′), 126.6 (CH, C-14), 127.5 (C, C-6′), 140.1 (CH, C-13), 145.5 (C, C-1′), 146.0 (C, C-5).

### 4.9. Statistical Analysis

The data were expressed as means ± standard deviation. Statistical analysis of raw data was performed using the software JMP^®^ version 19 (2025, JMP Statistical Discovery LLC., Cary, NC, USA). Analysis of variance (one-way ANOVA), followed by Dunnett’s and Tukey’s multiple pairwise comparison post-test, was performed to assess significant differences between treatments and the control sample. Log_2_-transformed data of antimicrobial activity were used for statistical analysis to comply with the assumptions of ANOVA and Tukey’s test. Correlations among the three target strains of foodborne pathogens were also assessed. The response variables tested were antimicrobial activity against the foodborne pathogens *L. monocytogenes*, *S. aureus* (MIC) and *B. cereus* (MBC), and antioxidant activity (IC_50_) based on the DPPH assay, together with TPC and carbohydrate, fucoxanthin, chlorophyll A, protein, and phloroglucinol contents.

## 5. Conclusions

The overall findings underscore the importance of accounting for seasonal and interannual environmental variability when selecting macroalgal biomass for the production of bioactive extracts, whereas the effect of pretreatment was minimal.

Chemical characterization by NMR revealed the presence of several bioactive compounds, with phloroglucinol identified as one of the main secondary metabolites, alongside mannitol and alanine. Additionally, a mixture of meroditerpenoids was detected, with bifurcarenone and demethoxy cystoketalchromane identified as the predominant compounds. Pigments such as chlorophyll and fucoxanthin, as well as residual carbohydrate fractions, were also present in the crude extracts.

Despite these findings, a direct relationship between antioxidant and antimicrobial activities and individual bioactive components such as polyphenols, phloroglucinol, carbohydrates, proteins, chlorophyll, and fucoxanthin could not be fully established. Fractionation of the crude extract indicated that a meroterpenoid-rich fraction exhibited markedly enhanced antimicrobial activity but reduced antioxidant activity, suggesting that meroterpenoids could have an important role in antimicrobial activity, whereas antioxidant activity is likely associated with other compounds diminished during fractionation, such as mannitol, phloroglucinol, and chlorophyll A. Therefore, future studies should focus on the isolation and individual evaluation of these compounds to clarify their specific contributions and to achieve a more comprehensive understanding of the bioactivity mechanisms of *E. selaginoides* extracts.

## Figures and Tables

**Table 1:** Ratio of signal intensities of 1H NMR spectra in D_2_O and CD_3_OD.

Extracts	^a^ δ_H_ 6.00/3.86/1.47	^b^ δ_H_ 5.79/3.80
April 2022 control	3/35.50/1.44	3/5.36
April 2022 treated (HHP 600 MPa for 5 min)	3/49.92/1.81	3/11.45
September 2022 control	3/76.52/14.19	3/9.47
September 2022 treated (HHP 600 MPa for 5 min)	3/79.09/5.51	3/14.45

^a^ D_2_O; ^b^ CD_3_OD.

## Data Availability

The data presented in this study are available in this article and the Supplementary Materials; further inquiries can be directed to the corresponding authors.

## Supplementary Materials

The following supporting information can be downloaded at https://www.mdpi.com/article/10.3390/md24080257/s1. Table S1. Seasonal variations in extract yield (g) of the control (fresh) and HHP treated extracts (600 MPa for 5 min) of *E. selaginoides*. Table S2. Seasonal variations of antimicrobial activity in the control (fresh) and treated HHP (600 MPa for 5 min) extracts of *E. selaginoides*. Table S3. Seasonal variations in antioxidant activity (measured by 50% inhibition concentration (IC_50_) in the control (fresh) and treated HHP (600 MPa for 5 min) extracts of *E. selaginoides*. Table S4. Monthly and seasonal variation of macronutrients, carbohydrate and protein contents (µg/g dry wt) in extracts of *E. selaginoides*. Table S5. Monthly and seasonal variation of pigments, chlorophyll A and fucoxanthin contents (µg/g dry wt) in extracts of *E. selaginoides*. Table S6. Seasonal variations in total polyphenol contents TPC (µg/g dry wt) in the fresh (control) and treated HHP (600 MPa for 5 min) extracts of *E. selaginoides*. Table S7. Seasonal variations in phloroglucinol contents (µg/g dry wt) in the fresh (control) and HHP pretreated 600 MPa for 5 min) extracts of *E. selaginoides*. Figure S1. ^1^H NMR spectrum of crude extract in D_2_O. Figure S2. ^13^C NMR spectrum of crude extract in D_2_O. Figure S3. ^1^H NMR spectrum of crude extract in CD_3_OD. Figure S4. ^13^C NMR spectrum of crude extract in CD_3_OD. Figure S5. ^1^H NMR spectrum in D_2_O of control extract April 2022 showing integral of phloroglucinol δ_H_ 6.00 ppm mannitol δ_H_ 3.86 ppm and alanine δ_H_ 1.47 ppm signals. Figure S6. ^1^H NMR spectrum in D_2_O of HHP 600 MPa for 5 extract April 2022 showing integral of phloroglucinol δ_H_ 6.00 ppm, mannitol δ_H_ 3.86 ppm alanine δ_H_ 1.47 ppm signals. Figure S7. ^1^H NMR spectrum in D_2_O of control extract September 2022 showing integral of phloroglucinol δ_H_ 6.00 ppm, mannitol δ_H_ 3.86 ppm alanine δ_H_ 1.47 ppm signals. Figure S8. ^1^H NMR spectrum in D_2_O of HHP 600 MPa for 5 extract September 2022 showing integral of phloroglucinol δ_H_ 6.00 ppm, mannitol δ_H_ 3.86 ppm alanine δ_H_ 1.47 ppm signals. Figure S9. ^1^H NMR spectrum in CD_3_OD of control extract April 2022 showing integral of phloroglucinol δ_H_ 5.79 ppm and mannitol δ_H_ 3.80 ppm signals. Figure S10. ^1^H NMR spectrum in CD_3_OD of HHP 600 MPa for 5 extract April 2022 showing integral of phloroglucinol δ_H_ 5.79 ppm and mannitol δ_H_ 3.80 ppm signals. Figure S11. ^1^H NMR spectrum in CD_3_OD of control extract September 2022 showing integral of phloroglucinol δ_H_ 5.79 ppm and mannitol δ_H_ 3.80 ppm signals. Figure S12. ^1^H NMR spectrum in CD_3_OD of HHP 600 MPa for 5 extract September 2022 showing integral of phloroglucinol δ_H_ 5.79 ppm and mannitol δ_H_ 3.80 ppm signals. Figure S13. ^1^H NMR spectrum of F2 fraction in CD_3_OD. Figure S14. ^13^C NMR spectrum of F2 fraction in CD_3_OD. Figure S15. COSY NMR spectrum of F2 fraction in CD_3_OD. Figure S16. HSQC NMR spectrum of F2 fraction in CD_3_OD. Figure S17. HMBC NMR spectrum of F2 fraction in CD_3_OD. Figure S18. ^1^H NMR spectrum of F2 fraction in CDCl_3_. Figure S19. ^13^C NMR spectrum of F2 fraction in CDCl_3_. Figure S20. COSY NMR spectrum of F2 fraction in CDCl_3_. Figure S21. HSQC NMR spectrum of F2 fraction in CDCl_3_. Figure S22. HMBC NMR spectrum of F2 fraction in CDCl_3_.
