# Supplementary material for: Seasonality: The Driving Force Behind the Antimicrobial and Antioxidant Activities and Biochemical Composition of Ericaria selaginoides Extracts, with Minimal Effect of High Hydrostatic Pressure Pretreatment
Source: Mar Drugs. 2026 Jul 25;24(8):257. doi: 10.3390/md24080257 (PMC13514295; doi:10.3390/md24080257)

SUPPLEMENTARY MATERIALS

Seasonality: the Driving Force Behind the Antimicrobial and Antioxidant Activities and Biochemical Composition of *Ericaria selaginoides* Extracts, with Minimal Effect of High-Hydrostatic-Pressure Pretreatment

Sunuram Ray[1,2], Maria Hortos[1,†], Andrea Casal-Silva[3], Mercedes Cueto[3,*], and Teresa Aymerich[1,*].

1 Food Safety and Functionality Program, Institute of Agrifood Research and Technology (IRTA), 17121 Monells, Girona (Spain); sunuram.ray@irta.cat

2 Institute for Integrated Studies on the Sundarbans and Coastal Ecosystems (IISSCE), Khulna University, Khulna-9208, Bangladesh

3 Institute of Natural Products and Agrobiology (IPNA-CSIC), 38206 La Laguna, Tenerife, Spain; andreacasal@ipna,csic.es

Correspondence: teresa.aymerich@irta.cat; mcueto@ipna.csic.es

† Deceased, 3 March, 2026

Contents

Table S1. Seasonal variations in extract yield (g) of the control (fresh) and HHP treated extracts (600 MPa for 5 min) of *E. selaginoides*. 1

Table S2. Seasonal variations of antimicrobial activity in the control (fresh) and treated HHP (600 MPa for 5 min) extracts of *E. selaginoides*........ 2

Table S3. Seasonal variations in antioxidant activity (measured by 50% inhibition concentration (IC_{50}) in the control (fresh) and treated HHP (600 MPa for 5 min) extracts of *E. selaginoides*. 3

Table S4. Monthly and seasonal variation of macronutrients, carbohydrate and protein contents (µg/g dry wt) in extracts of *E. selaginoides*........ 3

Table S5. Monthly and seasonal variation of pigments, chlorophyll A and fucoxanthin contents (µg/g dry wt) in extracts of *E. selaginoides*........ 3

Table S6. Seasonal variations in total polyphenol contents TPC (µg/g dry wt) in the fresh (control) and treated HHP (600 MPa for 5 min) extracts of *E. selaginoides*. 4

Table S7. Seasonal variations in phloroglucinol contents (µg/g dry wt) in the fresh (control) and HHP pretreated 600 MPa for 5 min) extracts of *E. selaginoides*. 4

Figure S1. ^{1}H NMR spectrum of crude extract in D_2O 5

Figure S2. ^{13}C NMR spectrum of crude extract in D_2O........ 5

Figure S3. ^{1}H NMR spectrum of crude extract in CD_3OD 6

Figure S4. ^{13}C NMR spectrum of crude extract in CD_3OD 6

Figure S5. ^1H NMR spectrum in D_2O of extract April 2022 showing integral of phloroglucinol δ_H 6.00 ppm, mannitol δ_H 3.86 ppm alanine δ_H 1.47 ppm signals 7

Figure S6. ^1H NMR spectrum in D_2O of HHP 600MPa for 5 extract April 2022 showing integral of phloroglucinol δ_H 6.00 ppm, mannitol δ_H 3.86 ppm alanine δ_H 1.47 ppm signals 7

Figure S7. ^1H NMR spectrum in D_2O of control extract September 2022 showing integral of phloroglucinol δ_H 6.00 ppm, mannitol δ_H 3.86 ppm alanine δ_H 1.47 ppm signals 8

Figure S8. ^1H NMR spectrum in D_2O of HHP 600MPa for 5 extract September 2022 showing integral of phloroglucinol δ_H 6.00 ppm, mannitol δ_H 3.86 ppm alanine δ_H 1.47 ppm signals 8

Figure S9. ^1H NMR spectrum in CD_3OD of control extract April 2022 showing integral of phloroglucinol δ_H 5.79 ppm and mannitol δ_H 3.80 ppm signals 9

Figure S10. ^1H NMR spectrum in CD_3OD of HHP 600MPa for 5 extract April 2022 showing integral of phloroglucinol δ_H 5.79 ppm and mannitol δ_H 3.80 ppm signals 10

Figure S11. ^1H NMR spectrum in CD_3OD of control extract September 2022 showing integral of phloroglucinol δ_H 5.79 ppm and mannitol δ_H 3.80 ppm signals 10

Figure S12. ^1H NMR spectrum in CD_3OD of HHP 600MPa for 5 extract September 2022 showing integral of phloroglucinol δ_H 5.79 ppm and mannitol δ_H 3.80 ppm signals 11

Figure S13. ^1H NMR spectrum of F2 fraction in CD_3OD 12

Figure S14. ^{13}C NMR spectrum of F2 fraction in CD_3OD 12

Figure S15. COSY NMR spectrum of F2 fraction in CD_3OD 13

Figure S16. HSQC NMR spectrum of F2 fraction in CD_3OD 13

Figure S17. HMBC NMR spectrum of F2 fraction in CD_3OD 14

Figure S18. ^1H NMR spectrum of F2 fraction in $CDCl_3$ 14

Figure S19. ^{13}C NMR spectrum of F2 fraction in $CDCl_3$ 15

Figure S20. COSY NMR spectrum of F2 fraction in $CDCl_3$ 15

Figure S21. HSQC NMR spectrum of F2 fraction in $CDCl_3$ 16

Figure S22. HMBC NMR spectrum of F2 fraction in $CDCl_3$ 16

Table S1. Seasonal variations in extract yield (g) of the control (fresh) and HHP treated extracts (600 MPa for 5 min) of *E. selaginoides*.

Recollection period	Month/year	Extract yield (g) in control (fresh)	Extract yield (g) in HHP (600 MPa for 5 min)
**Winter**	February 2022	0.23 ± 0.02	0.23 ± 0.02
**Spring**	April 2022	0.22 ± 0.02	0.22 ± 0.02
	April 2023	0.25 ± 0.02	0.27 ± 0.03
**Summer**	July 2022	0.20 ± 0.02	0.23 ± 0.02
	September 2022	0.21± 0.02	0.19 ± 0.02
	September 2023	0.19 ± 0.02	0.22 ± 0.03
**Autumn**	November 2021	0.21± 0.02	0.23± 0.02

Values are shown as means ± standard deviation (n = 3). No significance letters are shown as all groups are not statistically significant, (ANOVA, Tukey's test, ($p > 0.05$).

T**able S2.** Seasonal variations of antimicrobial activity in the control (fresh) and treated HHP (600 MPa for 5 min) extracts of *E. selaginoides*.

**Foodborne pathogens**		***L. monocytogenes***				***S. aureus***				***B. cereus***			
**Categories**		**Control (fresh)**		**HHP (600MPa for 5 min)**		**Control (fresh)**		**HHP (600MPa for 5 min)**		**Control (fresh)**		**HHP (600MPa for 5 min)**	
**Recollection period**	Month/year	MIC (mg/mL)	Log_2 MIC (μg/mL)	MIC (mg/mL)	Log_2 MIC (μg/mL)	MIC (mg/mL)	Log_2 MIC (μg/mL)	MIC (mg/mL)	Log_2 MIC (μg/mL)	MBC (mg/mL)	Log_2 MBC (μg/mL)	MBC (mg/mL)	Log_2 MBC (μg/mL)
**Winter**	February 2022	5.66 ± 3.05	12.26 ± 0.30^{abA}	4.25 ± 1.33	11.98 ± 0.20^{bA}	8.82 ± 0.00	13.11 ± 0.00^{abA}	6.29 ± 5.55	12.24 ± 1.26^{bcA}	6.59 ± 3.76	12.35 ± 1.22^{abA}	6.10 ± 4.72	12.12 ± 1.33^{abA}
	February 2023	16.38 ± 3.13	13.99 ± 0.61^{aA}	19.75 ± 0.00	14.27 ± 0.33^{aB}	33.00 ± 1.41	15.01 ± 0.06^{aA}	37.33 ± 4.62	15.18 ± 0.19^{aA}	6.79 ± 2.43	12.65 ± 0.60^{aA}	21.28 ± 0.00	14.38 ± 0.00^{aB}
**Spring**	April 2022	0.70 ± 0.46	9.23 ± 0.50^{cA}	0.54 ± 0.26	9.01 ± 0.41^{eA}	1.10 ± 1.14	9.56 ± 1.53^{cA}	1.04 ± 0.09	10.02 ± 0.12^{dA}	1.40 ± 0.92	10.23 ± 1.00^{bA}	0.30 ± 0.00	8.25 ± 0.00^{cA}
	April 2023	8.67 ± 3.13	13.01 ± 0.50^{abA}	2.15 ± 1.06	10.93 ± 0.29^{bcdB}	20.36 ± 1.20	14.31 ± 0.08^{aA}	6.78 ± 0.49	12.73 ± 011^{bB}	12.69 ± 10.32	13.10 ± 1.55^{abA}	7.40 ± 2.17	12.81 ± 0.40^{abA}
**Summer**	July 2022	2.07 ± 1.46	10.81 ± 0.61^{bcA}	1.15 ± 0.84	9.95 ± 0.29^{deA}	1.97 ± 0.40	10.93 ± 0.29^{bcA}	1.40 ± 0.28	10.44 ± 0.29^{cdA}	4.82 ± 0.00	12.23 ± 0.00^{abA}	1.40 ± 0.23	10.44 ± 0.24^{bcB}
	September 2022	2.18 ± 0.82	11.01 ± 0.38^{bcA}	1.75 ± 0.61	10.71 ± 0.29^{cdA}	5.42 ± 5.24	11.95 ± 1.71^{abcA}	3.75 ± 1.01	11.83 ± 0.42^{bcA}	7.67 ± 7.17	12.41 ± 1.35^{abA}	2.68 ± 1.70	11.19 ± 0.95^{bcA}
	September 2023	7.99 ± 7.04	12.48 ± 0.43^{abA}	3.53 ± 1.45	11.69 ± 0.29^{bcA}	8.12 ± 1.41	12.98 ± 0.25^{abA}	5.04 ± 0.70	12.29 ± 0.20^{bcA}	14.13 ± 0.40	13.79 ± 0.04^{aA}	6.55 ± 2.67	12.60 ± 0.56^{abA}
**Autumn**	November 2021	2.97 ± 0.79	11.49 ± 0.43^{bA}	2.30 ± 0.17	11.17 ± 0.29^{bcdA}	15.34 ± 1.81	13.99 ± 0.16^{abA}	9.55 ± 0.83	13.22 ± 0.13^{bB}	2.43 ± 1.54	10.99 ± 1.02^{abA}	2.24 ± 1.94	10.66 ± 1.39^{bcA}

Values are shown as means ± standard deviation (*n* = 3). Here, min= minute, LM= *L. monocytogenes*, BC = *B. cereus*, SA = *S. aureus* and MIC = Minimum inhibitory concentration, MBC = Minimum bactericidal concentration. Different letters show statistical significance, p < 0. 05. Capital letter (A, B) shows treatment (HHP) comparison versus control in row for each target, small letter (a, b, c, d, e) shows comparison of seasons, years and months in column for each target. Statistical treatment was made for all the data together considering treatment and month and seasons

Table S3. Seasonal variations in antioxidant activity (measured by 50% inhibition concentration (IC_{50}) in the control (fresh) and treated HHP (600 MPa for 5 min) extracts of *E. selaginoides*.

Recollection period	Month/year	Control ((fresh)) IC_{50} (µg/mL)	HHP (600MPa for 5min) IC_{50} (µg/mL)	Average IC_{50} (µg/mL)
Winter	February 2022	237.66 ± 9.25^{abc}	273.43 ± 8.77^{a}	255.55 ± 9.01
Spring	April 2022	163.68 ± 15.86^{f}	169.60 ± 15.22^{ef}	166.64 ± 15.54
	April 2023	206.40 ± 20.23^{bcde}	223.55 ± 13.21^{bc}	214.98 ± 16.72
Summer	July 2022	174.88 ± 4.76^{ef}	202.04 ± 23.78^{cdef}	188.46 ± 14.27
	September 2022	170.80 ± 7.16^{ef}	178.81 ± 9.09^{def}	174.80 ± 8.13
	September 2023	209.93 ± 6.80^{bcde}	217.09 ± 21.79^{bcd}	213.51 ± 14.30
Autumn	November 2021	235.40 ± 3.72^{abc}	243.40 ± 11.39^{ab}	239.40 ± 7.56

Values are shown as means ± standard deviation (n = 3). Different letters show statistical significance, (ANOVA, Tukey's test, $p < 0.05$).

Table S4. Monthly and seasonal variation of macronutrients, carbohydrate and protein contents (µg/g dry wt) in extracts of *E. selaginoides*.

Recollection period	Month/year	Carbohydrate (µg/g dry wt)	Protein (µg/g dry wt)
Winter	February 2022	13,750.95 ± 2,884.84^{a}	ND
Spring	April 2022	11,886.43 ± 1,322.06^{ab}	ND
Summer	July 2022	10,667.55 ± 1,628.19^{abc}	4,323.69 ± 300.44
	September 2022	7,716.65 ± 257.99^{c}	ND
Autumn	November 2021	10,079.84 ± 573.47^{bc}	ND

Values are shown as means ± standard deviation (n = 3). Significant differences ($p < 0.05$) indicated by different superscript small letters. ND denote no detection of protein.

Table S5. Monthly and seasonal variation of pigments, chlorophyll A and fucoxanthin contents (µg/g dry wt) in extracts of *E. selaginoides.*

Recollection period	Month/year	Chlorophyll A (µg/g dry wt)	Fucoxanthin (µg/g dry wt)
Winter	February 2022	4,264.08 ± 600.18^{D}	4,072.78 ± 607.92^{c}
Spring	April 2022	9,877.01 ± 1,942.20^{A}	2,961.46 ± 289.48^{d}
Summer	July 2022	7,228.26 ± 1,211.60^{BC}	5,733.06 ± 5,522.80^{b}
	September 2022	9,463.31 ± 549.19^{AB}	7,916.07 ± 557.20^{a}
Autumn	November 2021	5,095 ± 638.60^{CD}	4,502.92 ± 171.35^{c}

Values are shown as means ± standard deviation (n = 3). Significant differences ($p < 0.05$) are indicated by capital different superscript letters for chlorophyll A and small letters for fucoxanthin.

Table S6. Seasonal variations in total polyphenol contents TPC (µg/g dry wt) in the fresh (control) and treated HHP (600 MPa for 5 min) extracts of *E. selaginoides*.

Recollection period	Month/year	TPC (µg/g dry wt) control (fresh)	TPC (µg/g dry wt) HHP (600MPa for 5min)	Average TPC (µg/g dry wt)
**Winter**	February 2022	9,187.32 ± 1,033.38^{de}	8,760.64 ± 857.66^{e}	8,973.98 ± 945.52
**Spring**	April 2022	15,691.36 ± 2,056.31^{b}	14,660.29 ± 1,813.97^{b}	15,175.83 ± 1,935.14
	April 2023	28,677.33 ± 693.89^{a}	29,291.37 ± 1,833.62^{a}	28,984 ± 1,263.76
**Summer**	July 2022	28,989.13 ± 1,865.18^{a}	27,813.17 ± 1,514.91^{a}	28,401.15 ± 1,690.05
	September 2022	12,193.06 ± 678.58^{c}	12,081.72 ± 432.91^{c}	12,137.39 ± 555.75
	September 2023	10,040.81 ± 1,149.91^{cde}	11,297.99 ± 1,735.12^{cd}	10,669.40 ± 1,442.52
**Autumn**	November 2021	9,546.23 ± 559.21^{de}	9,752.29 ± 828.27^{de}	9,649.26 ± 693.74

Values are shown as means ± standard deviation (n = 9). No significant difference between treated 600 MPa for 5 min and control extracts. Different letters show significance, $p < 0.05$.

Table S7. Seasonal variations in phloroglucinol contents (µg/g dry wt) in the fresh (control) and HHP pretreated 600 MPa for 5 min) extracts of *E. selaginoides*.

Recollection period	Month/year	Phloroglucinol (µg/g dry wt) at control (fresh)	Phloroglucinol (µg/g dry wt) at HHP (600MPa for 5min)	Average phloroglucinol (µg/g dry wt)
**Spring**	April 2022	166.39 ± 30.90^{a}	160.67 ± 19.51^{a}	163.53 ± 25.21^{a}
**Summer**	July 2022	172.77 ± 47.61^{a}	149.58 ± 11.88^{a}	161.18 ± 29.75^{a}
	September 2022	37.82 ± 8.84^{b}	47.06 ± 2.85^{b}	42.44 ± 5.85^{b}

Values are shown as means ± standard deviation (n = 3). Different superscript letters show significance, $p < 0.05$.

Figure S1. ^{1}H NMR spectrum of crude extract in D_2O

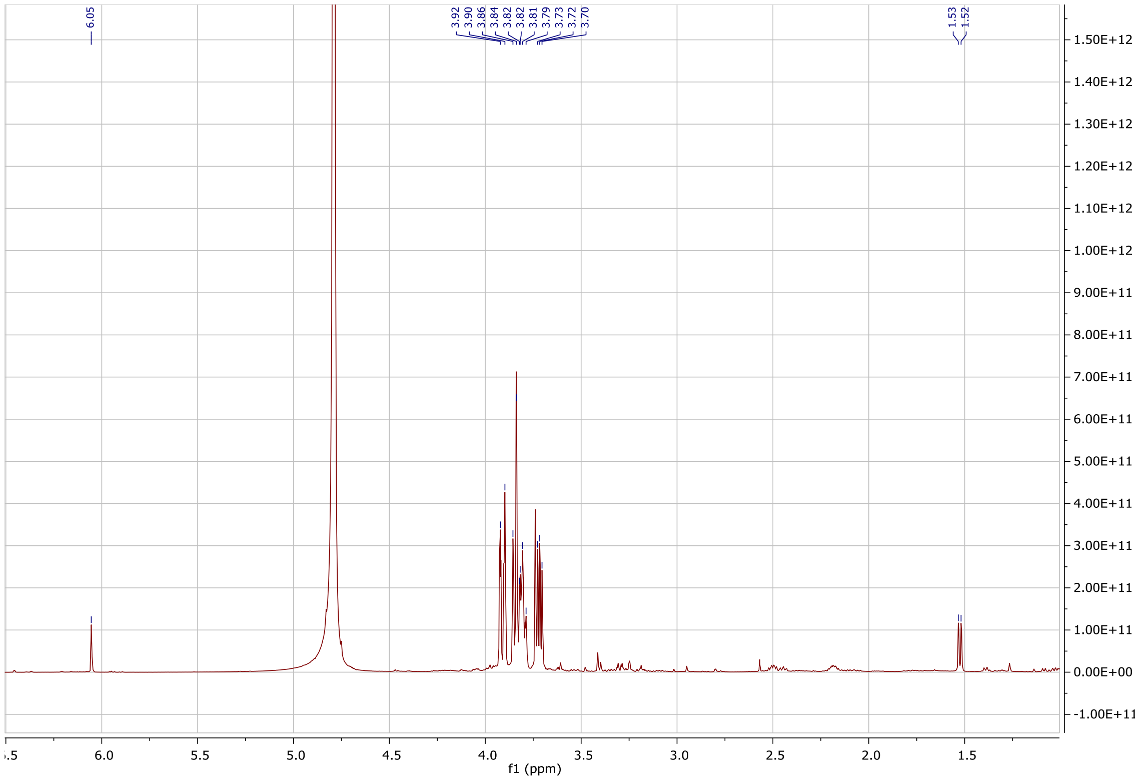

Figure S2. ^{13}C NMR spectrum of crude extract in D_2O

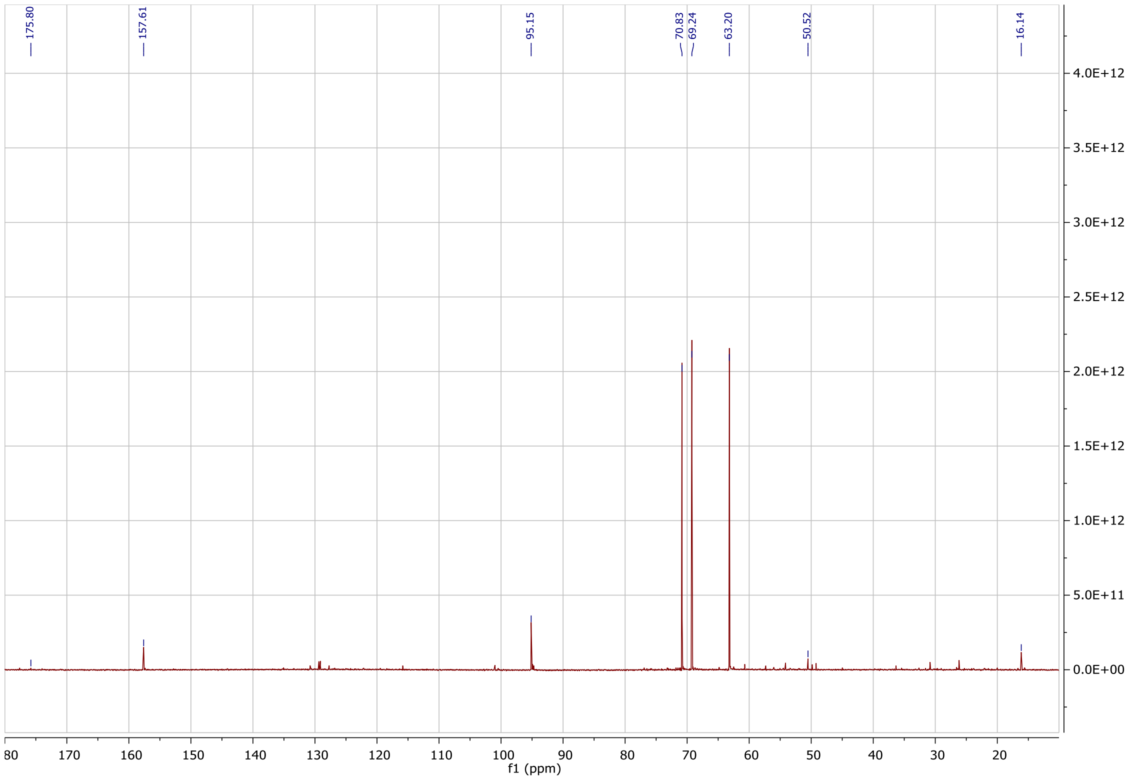

Figure S3. ^{1}H NMR spectrum of crude extract in CD_3OD

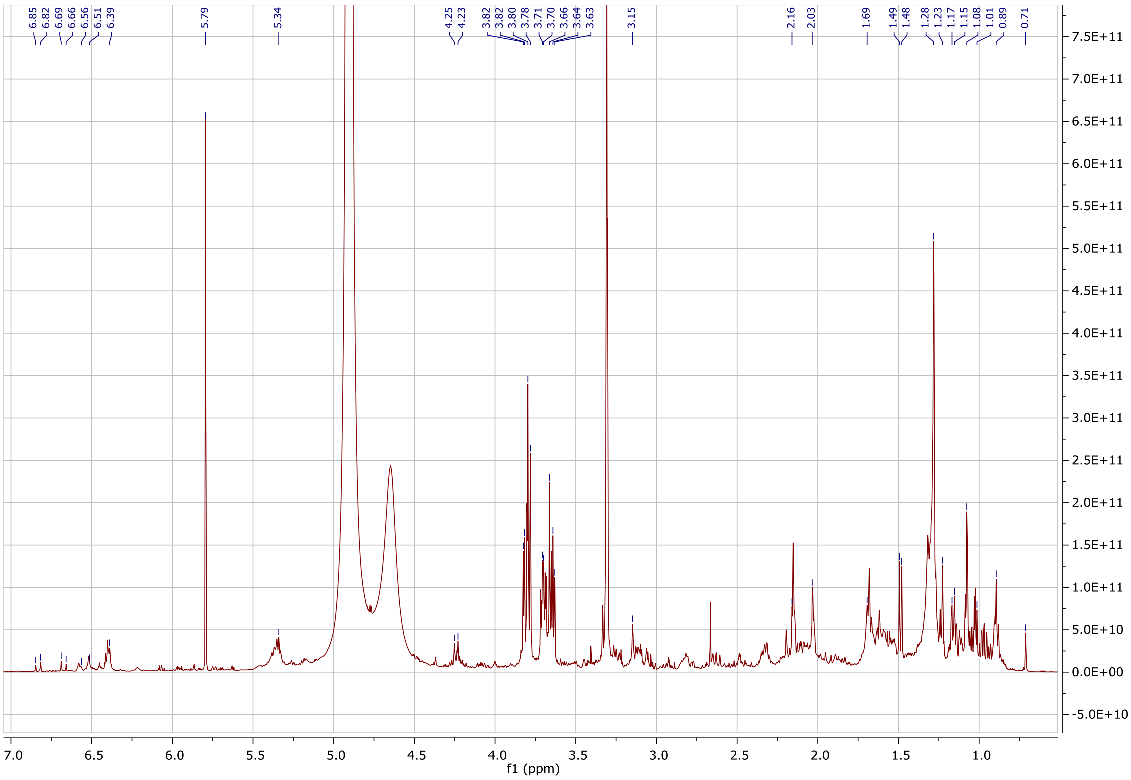

Figure S4. ^{13}C NMR spectrum of crude extract in CD_3OD

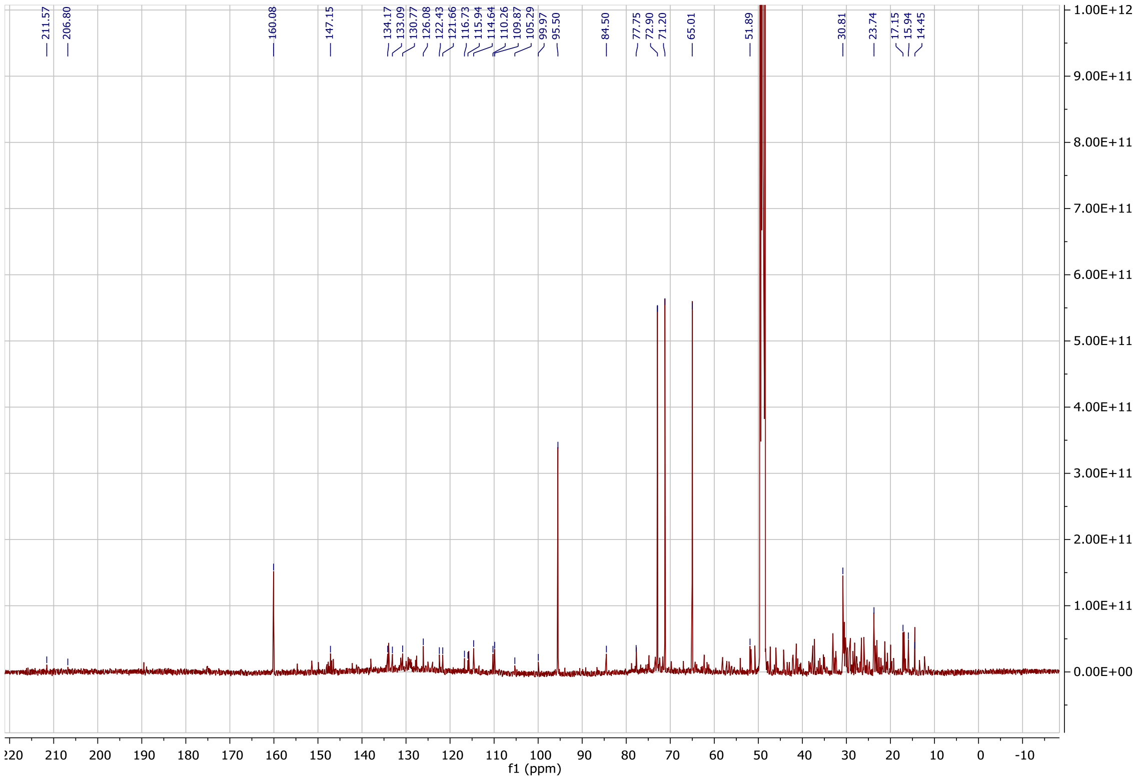

Figure S5. ^{1}H NMR spectrum in D_2O of control extract April 2022 showing integral of phloroglucinol δ_H 6.00 ppm mannitol δ_H 3.86 ppm and alanine δ_H 1.47 ppm signals.

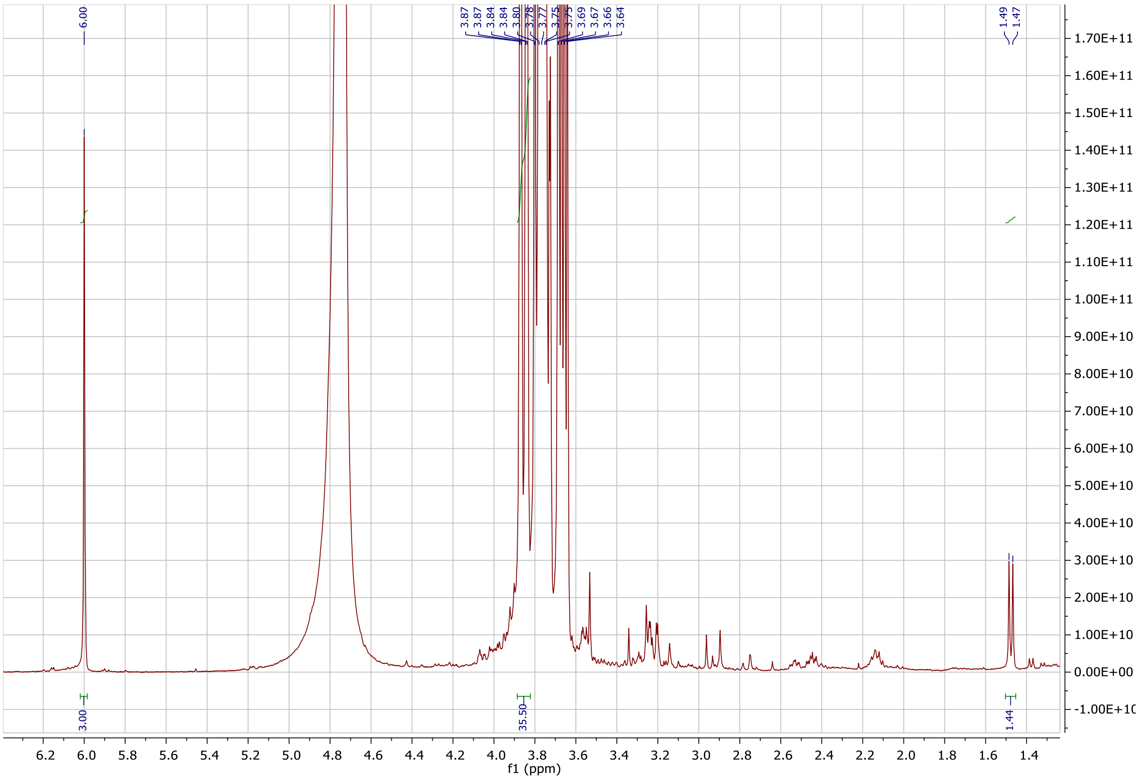

Figure S6. ^{1}H NMR spectrum in D_2O of HHP 600MPa for 5 extract April 2022 showing integral of phloroglucinol δ_H 6.00 ppm, mannitol δ_H 3.86 ppm alanine δ_H 1.47 ppm signals.

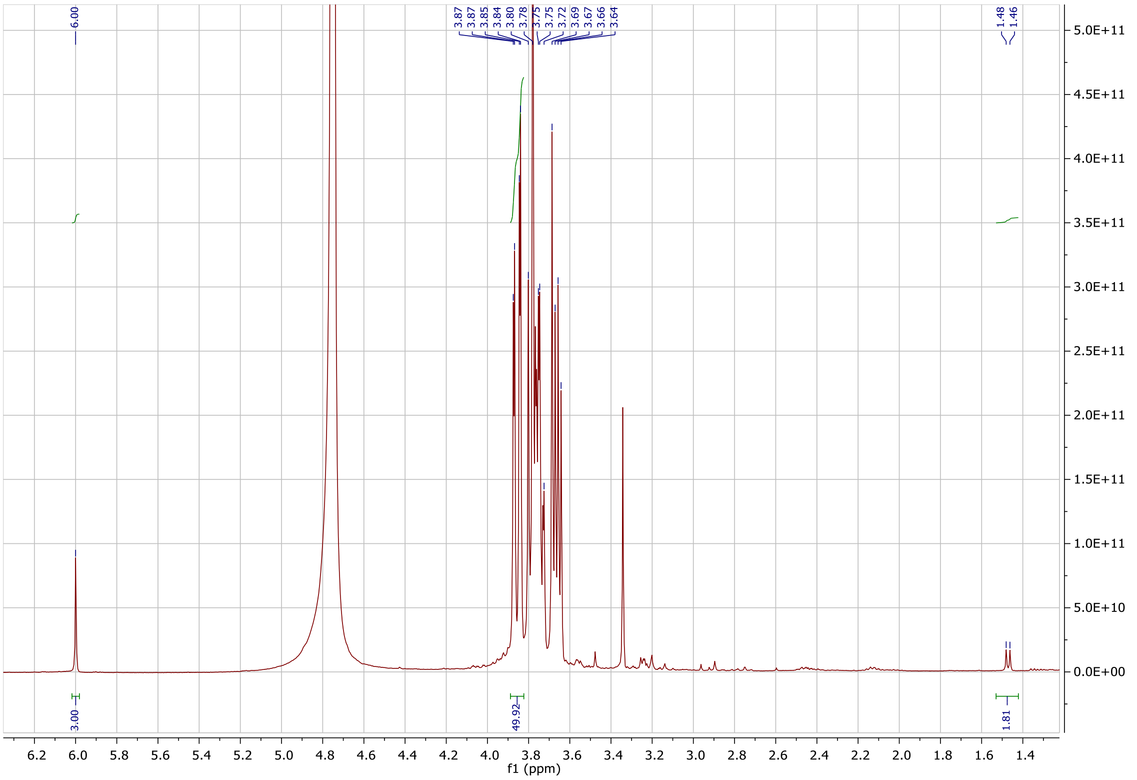

Figure S7. ^{1}H NMR spectrum in D_2O of control extract September 2022 showing integral of phloroglucinol δ_H 6.00 ppm, mannitol δ_H 3.86 ppm alanine δ_H 1.47 ppm signals.

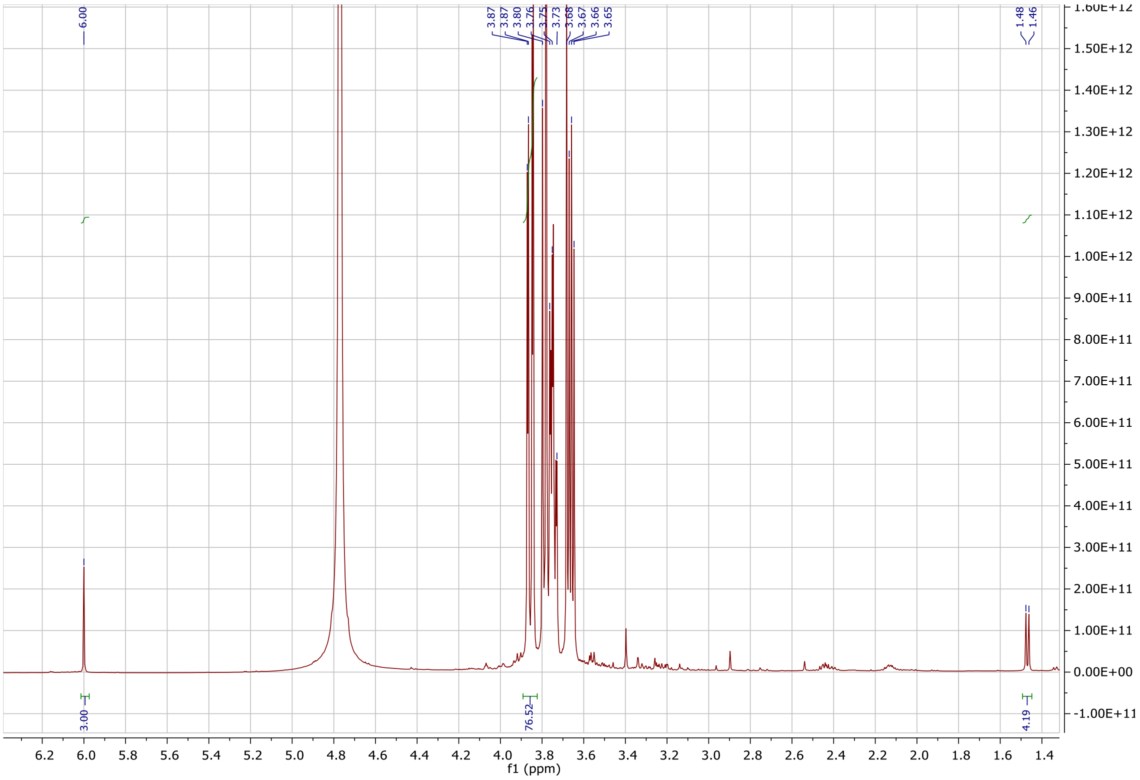

Figure S8. ^{1}H NMR spectrum in D_2O of HHP 600MPa for 5 extract September 2022 showing integral of phloroglucinol δ_H 6.00 ppm, mannitol δ_H 3.86 ppm alanine δ_H 1.47 ppm signals.

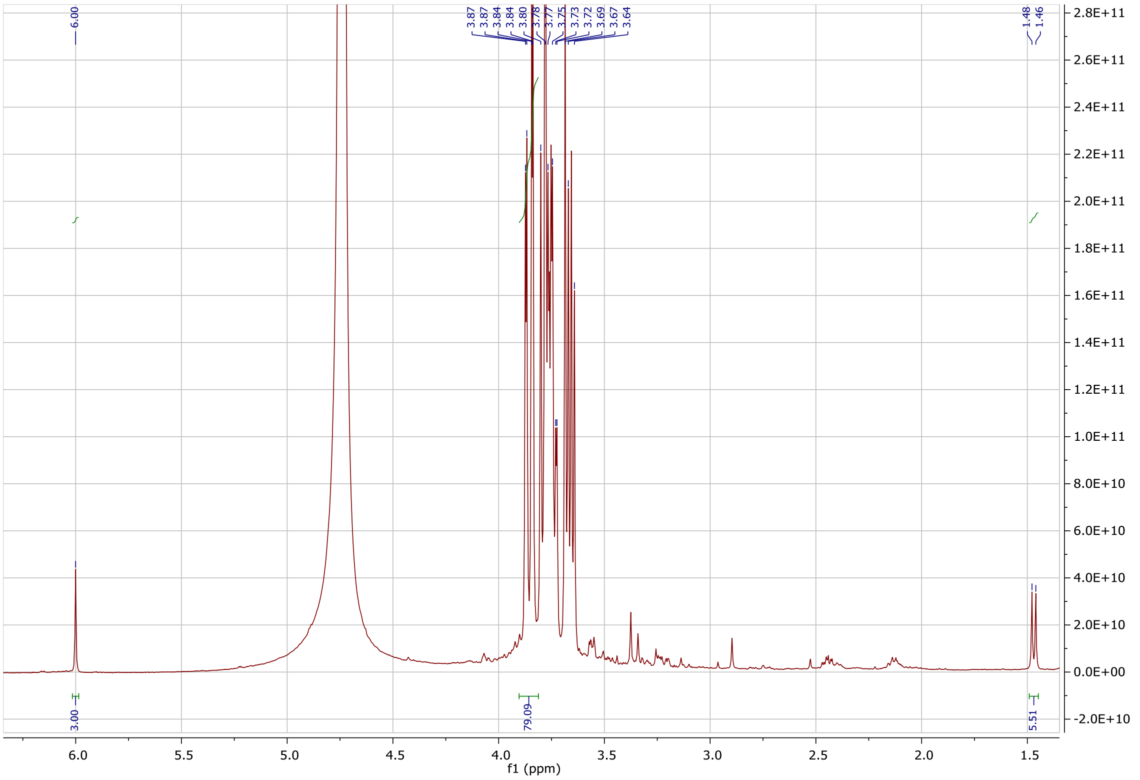

Figure S9. ^{1}H NMR spectrum in CD_3OD of control extract April 2022 showing integral of phloroglucinol δ_H 5.79 ppm and mannitol δ_H 3.80 ppm signals.

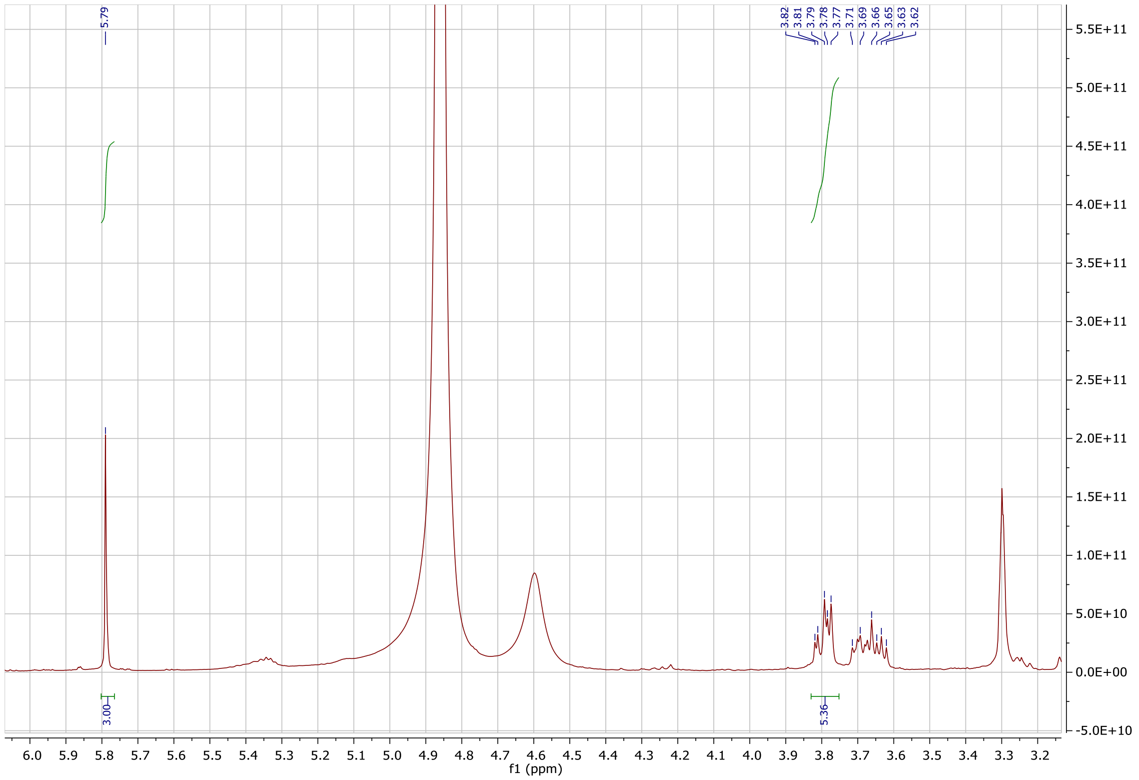

Figure S10. ^{1}H NMR spectrum in CD_3OD of HHP 600MPa for 5 extract April 2022 showing integral of phloroglucinol δ_H 5.79 ppm and mannitol δ_H 3.80 ppm signals.

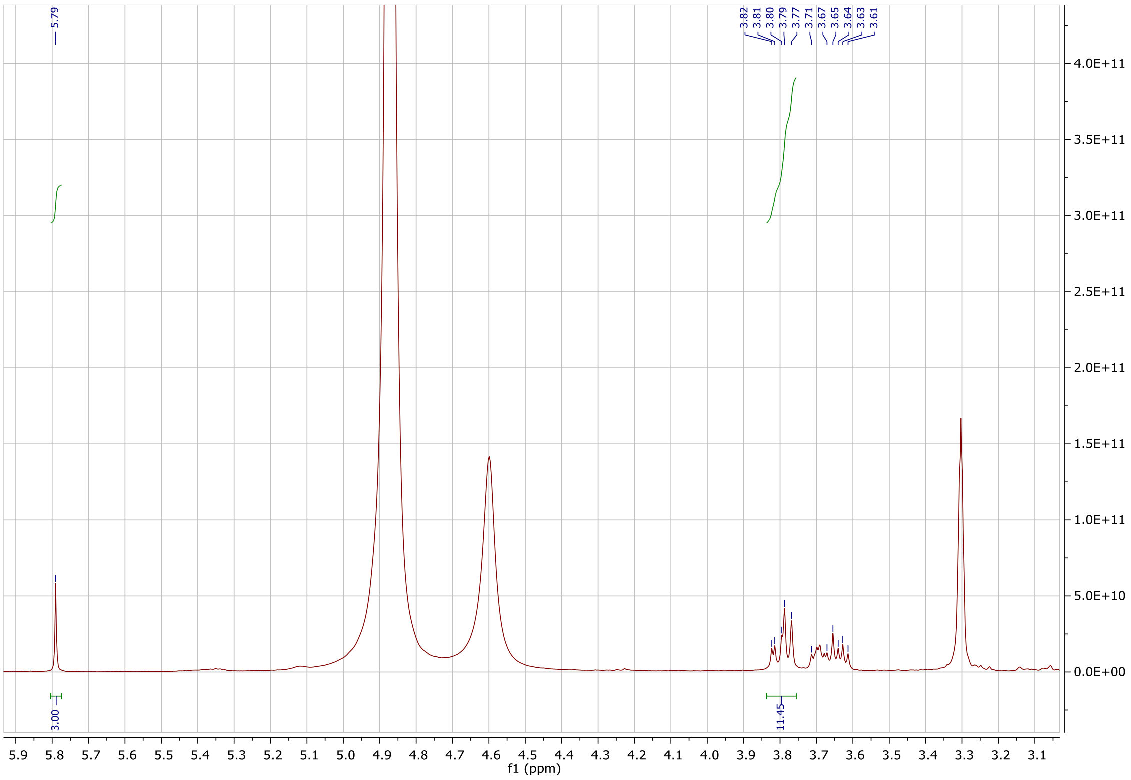

Figure S11. ^{1}H NMR spectrum in CD_3OD of control extract September 2022 showing integral of phloroglucinol δ_H 5.79 ppm and mannitol δ_H 3.80 ppm signals.

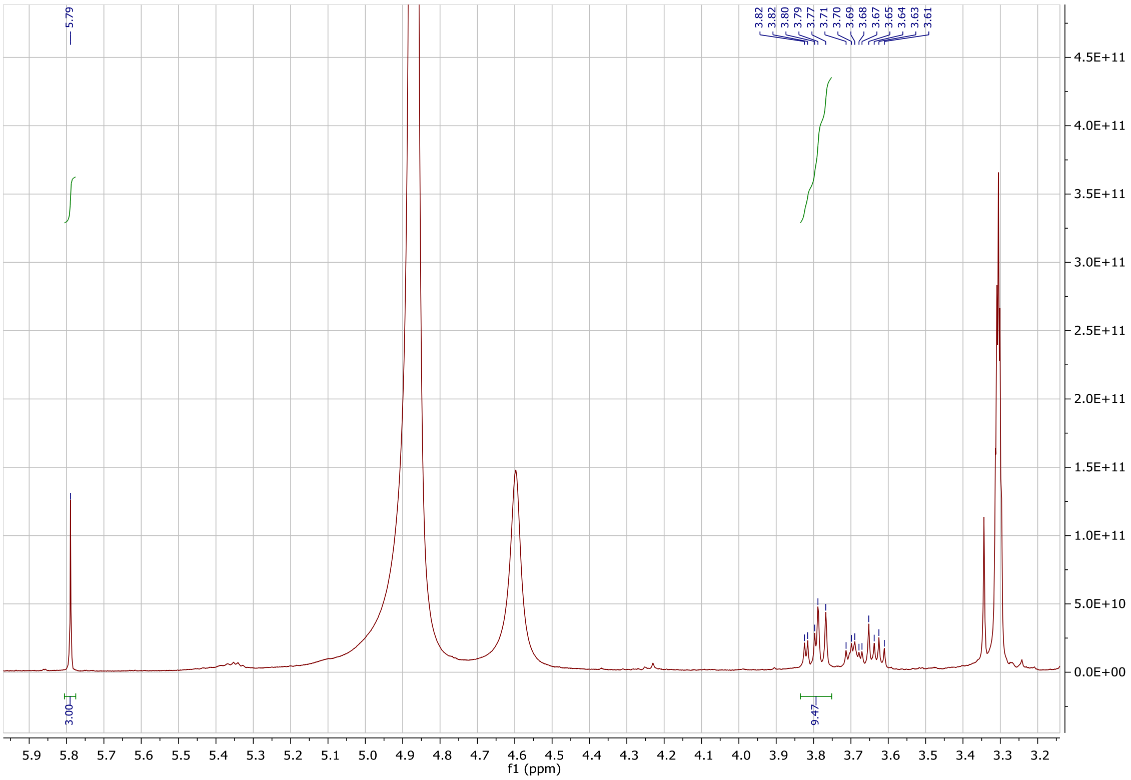

Figure S12. ¹H NMR spectrum in CD_3OD of HHP 600MPa for 5 extract September 2022 showing integral of phloroglucinol δ_H 5.79 ppm and mannitol δ_H 3.80 ppm signals.

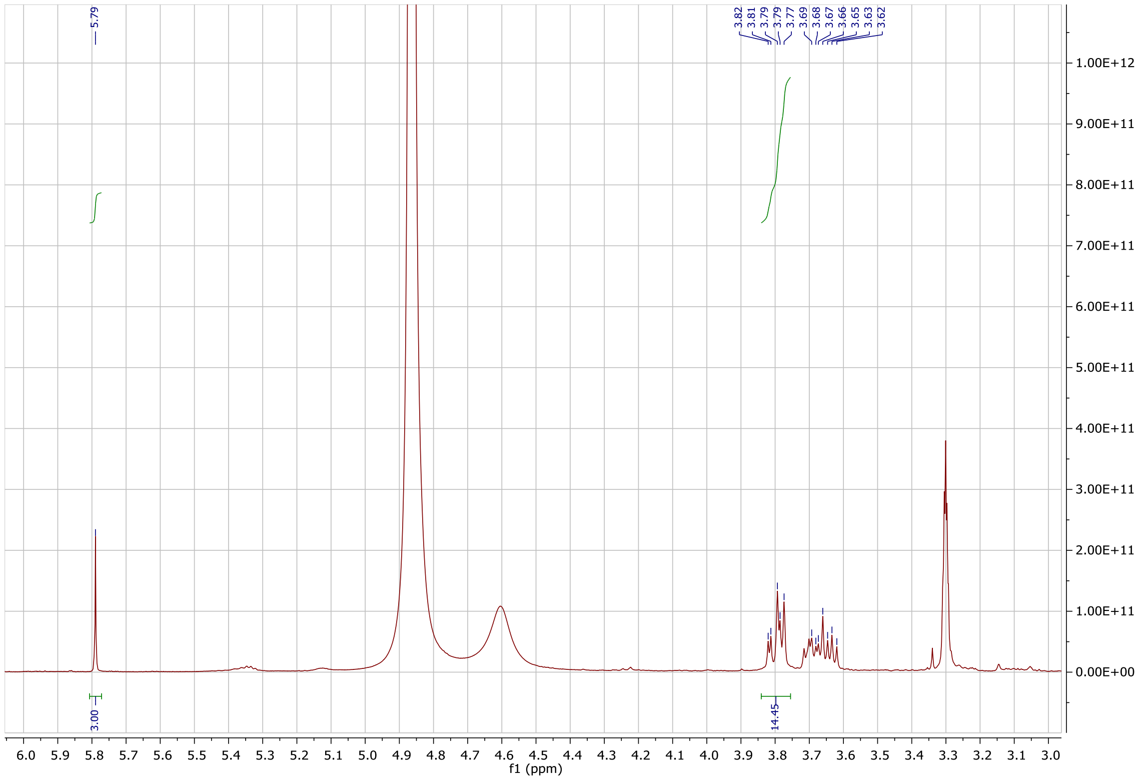

Figure S13. ^{1}H NMR spectrum of F2 fraction in CD_3OD

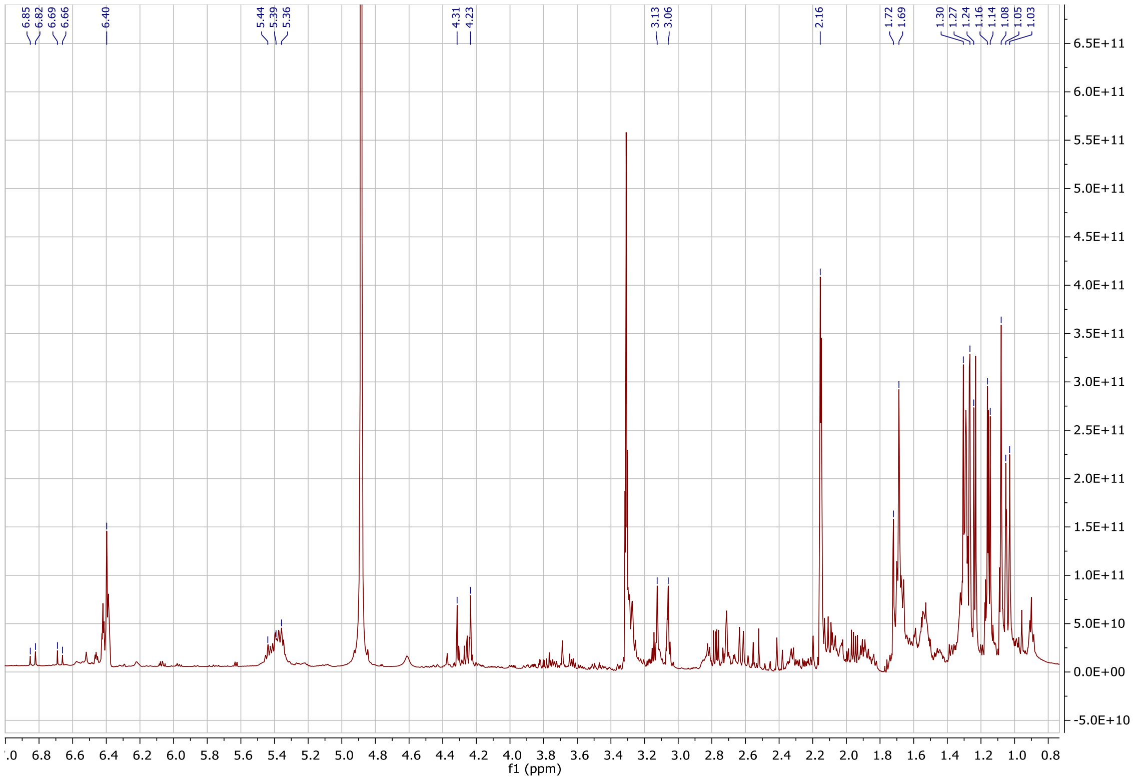

Figure S14. ^{13}C NMR spectrum of F2 fraction in CD_3OD

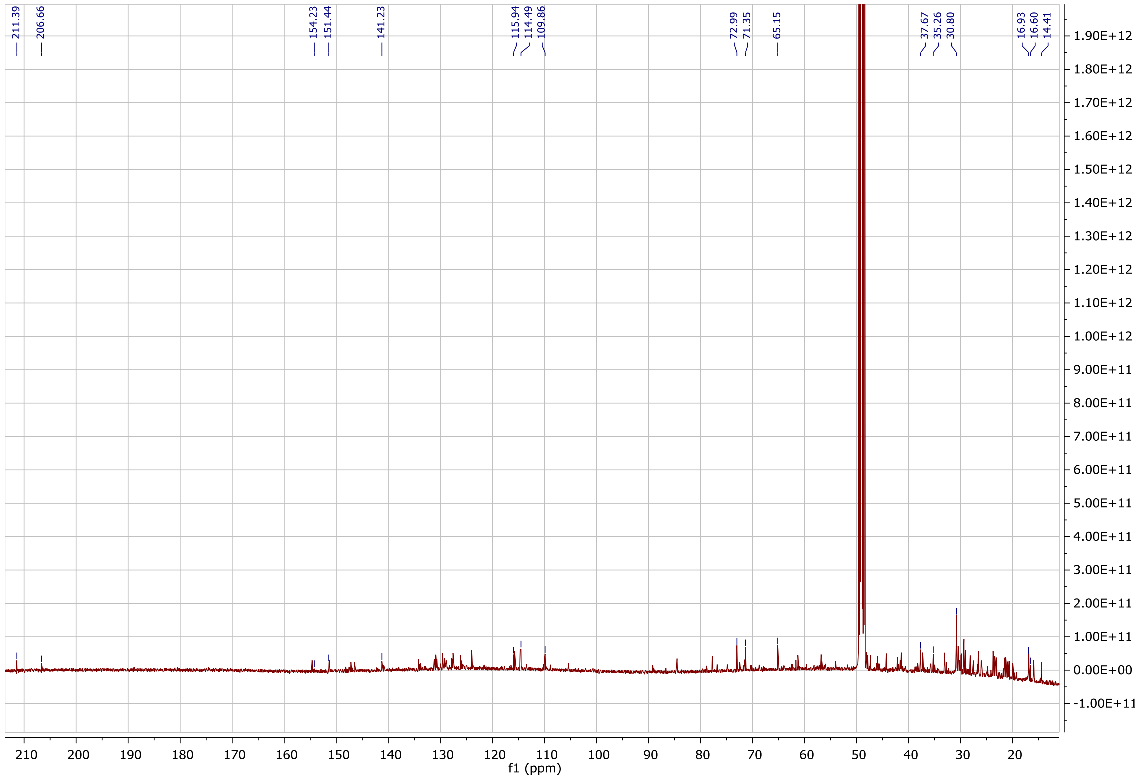

Figure S15. COSY NMR spectrum of F2 fraction in CD_3OD

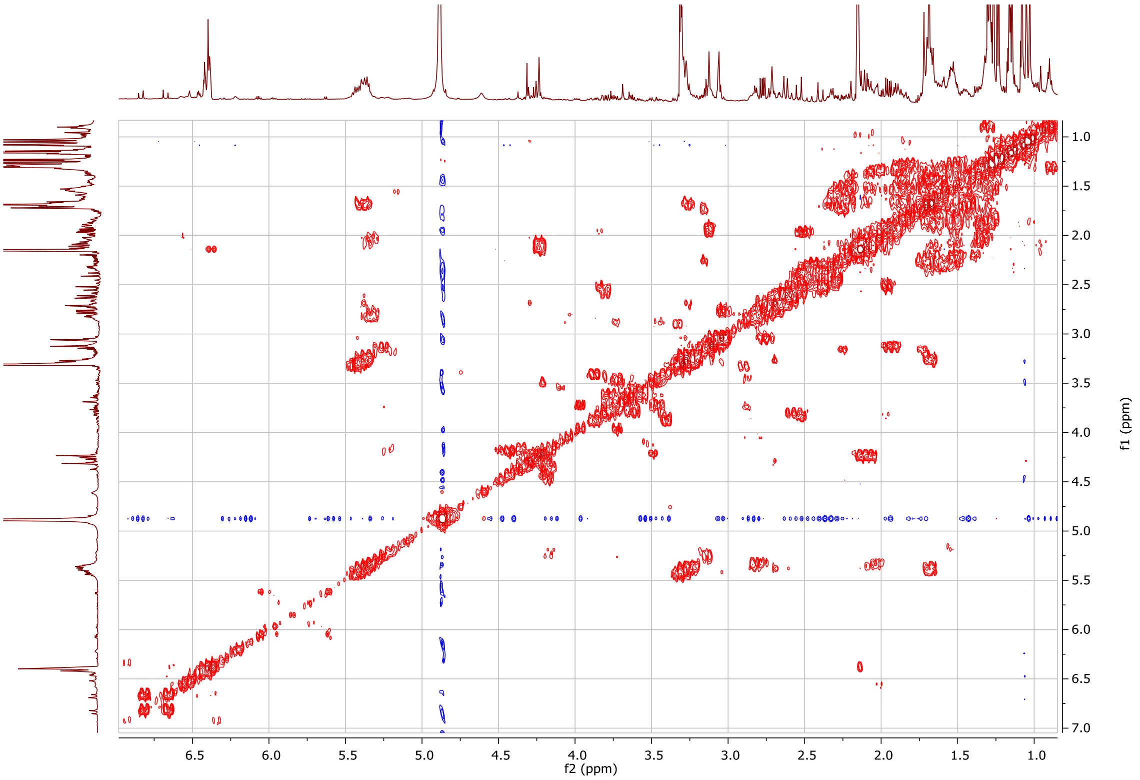

Figure S16. HSQC NMR spectrum of F2 fraction in CD_3OD

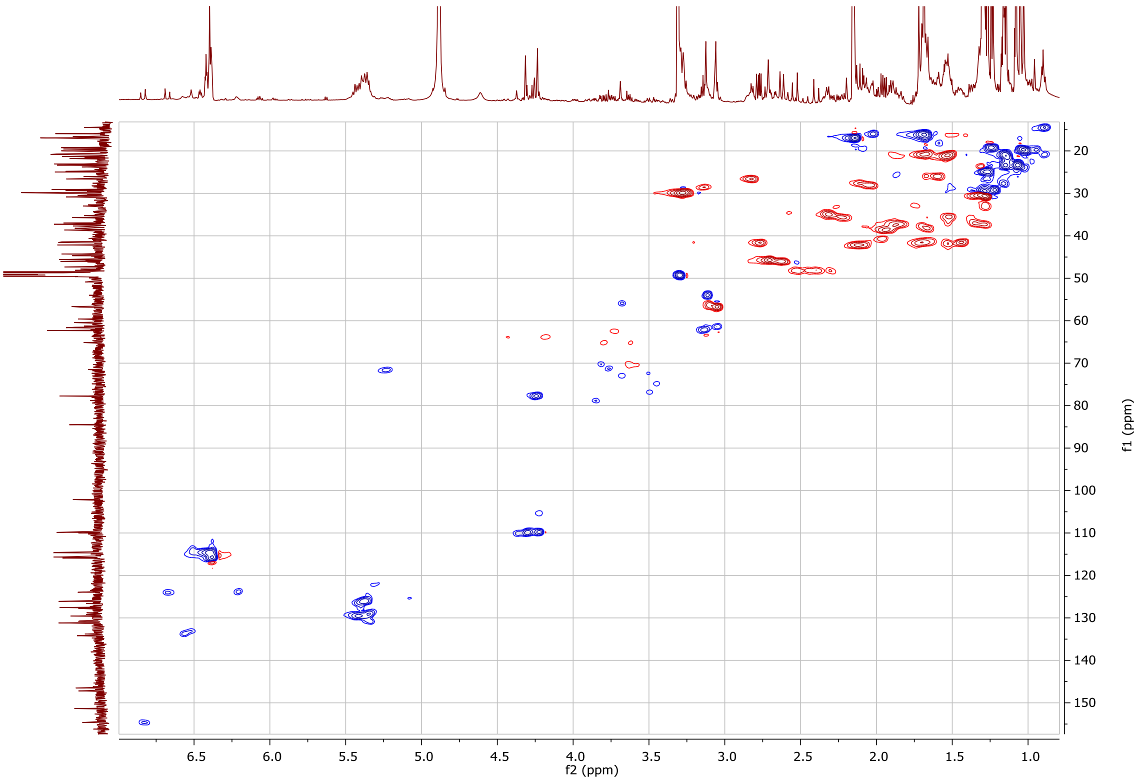

Figure S17. HMBC NMR spectrum of F2 fraction in CD_3OD

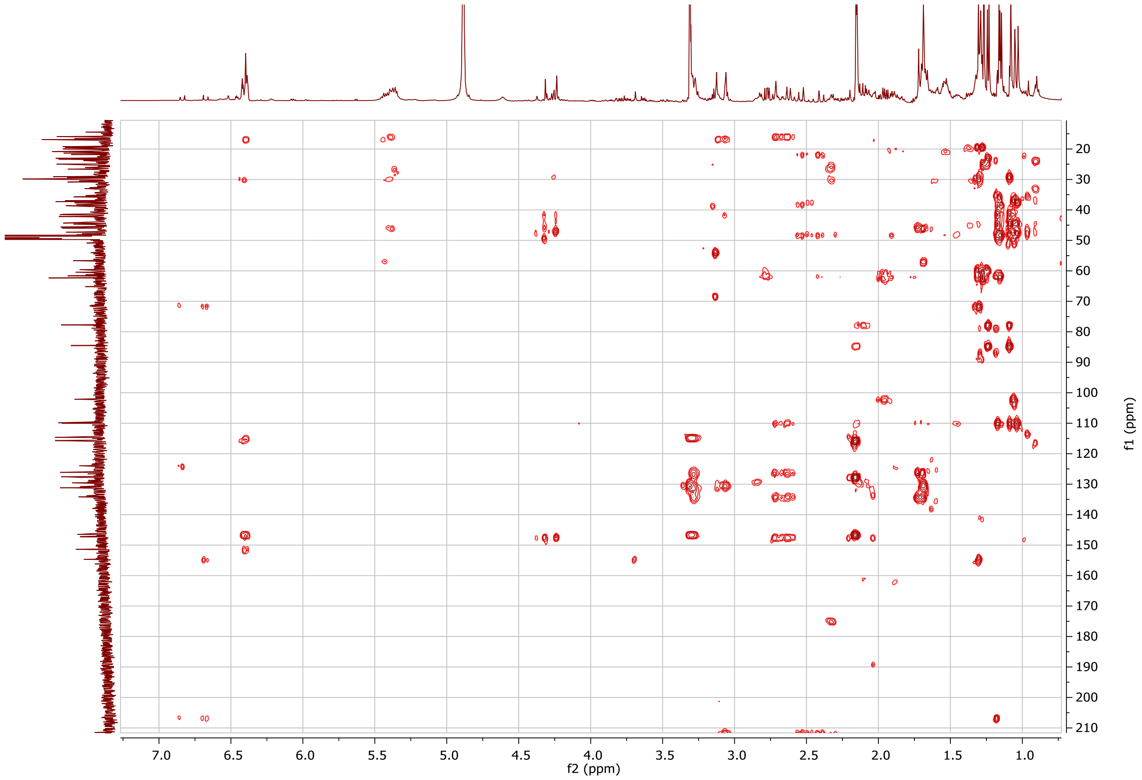

Figure S18. ^1H NMR spectrum of F2 fraction in $CDCl_3$

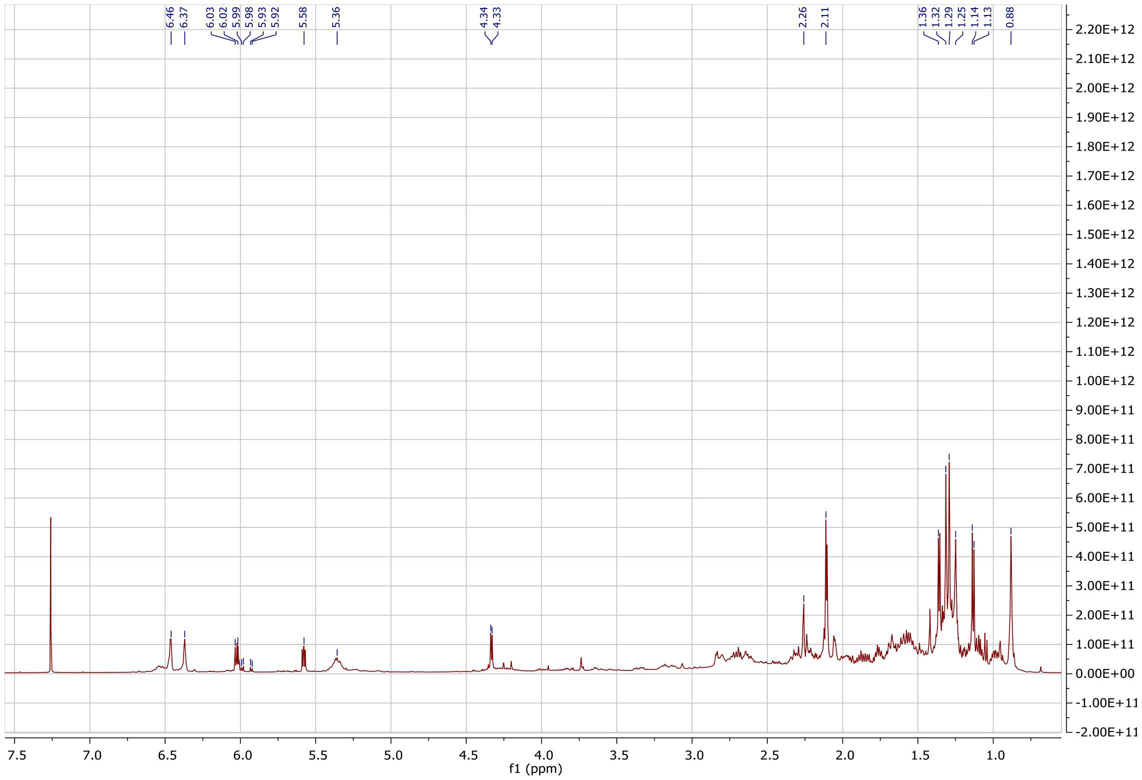

Figure S19. ^{13}C NMR spectrum of F2 fraction in $CDCl_3$

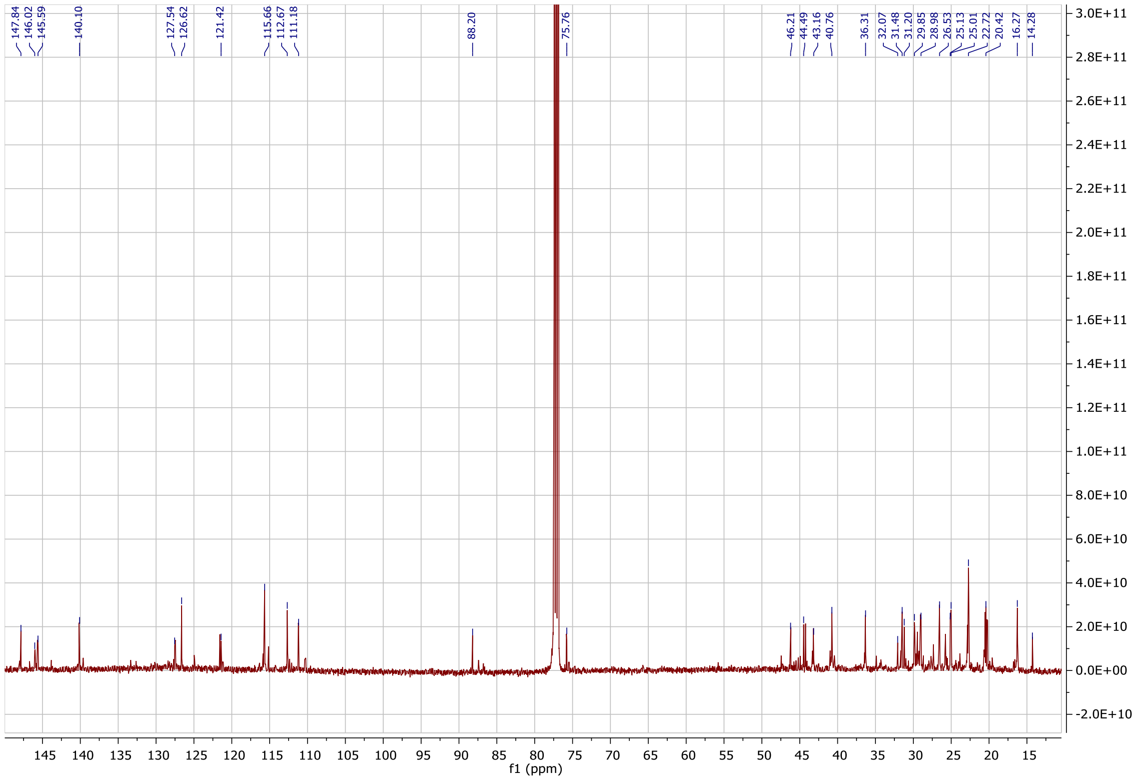

Figure S20. COSY NMR spectrum of F2 fraction in $CDCl_3$

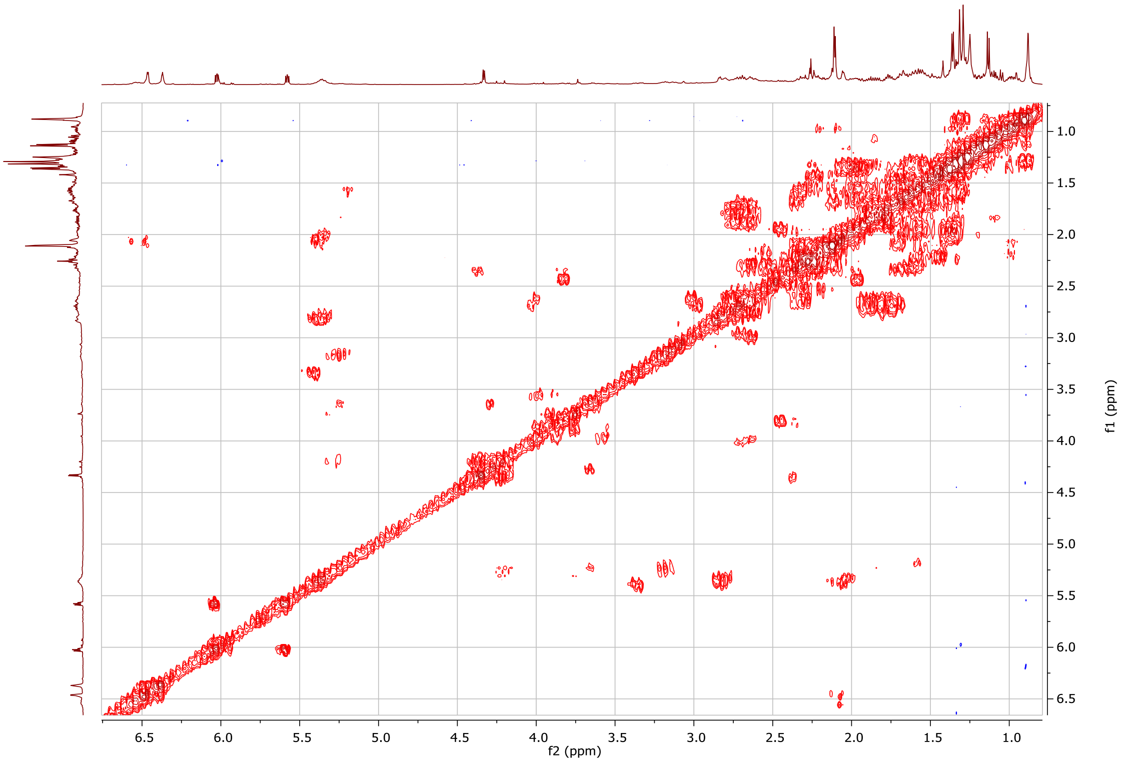

Figure S21. HSQC NMR spectrum of F2 fraction in CDCl3

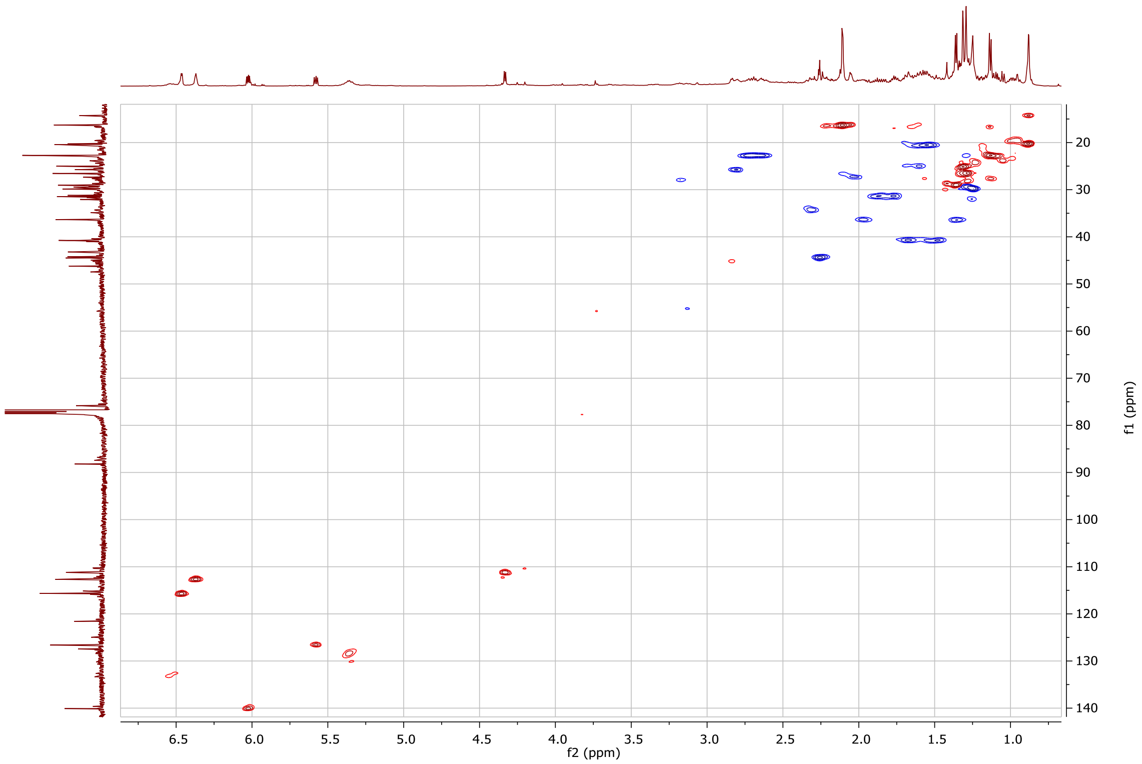

Figure S22. HMBC NMR spectrum of F2 fraction in CDCl3

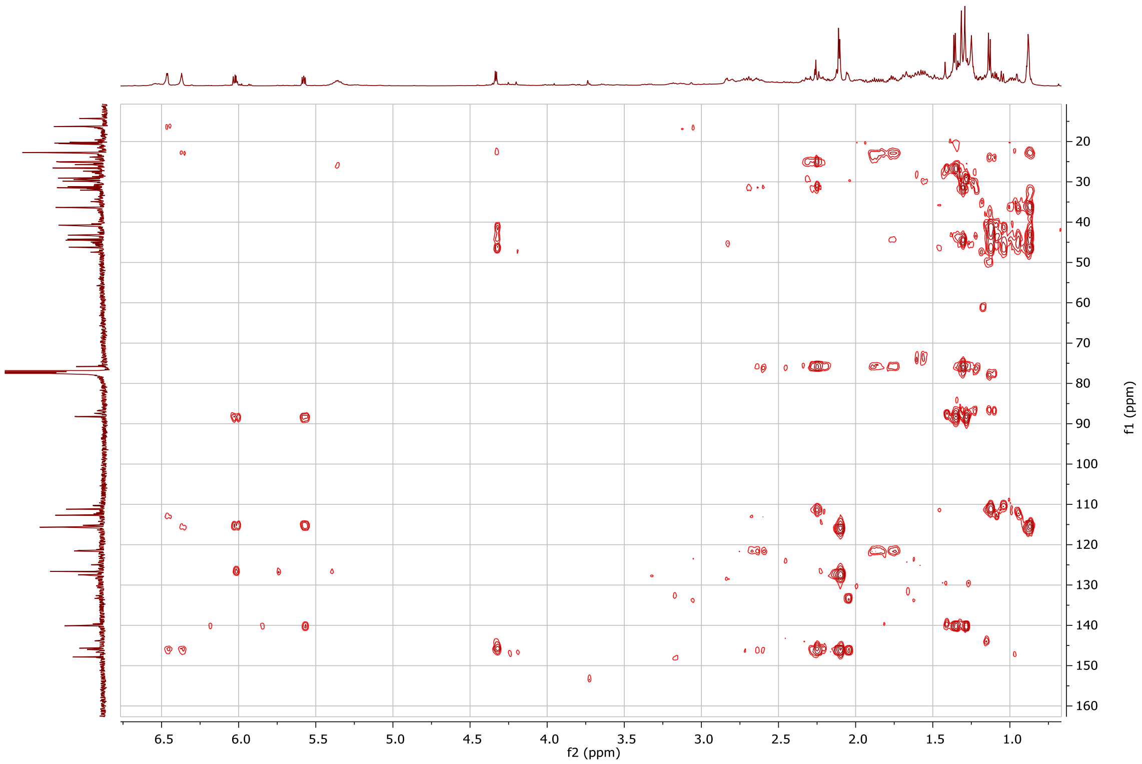

Supplement: Supplementary file 1 [file marinedrugs-24-00257-s001.zip › marinedrugs-4338498-supplementary.pdf]
